# An extended model for culture-dependent heterogenous gene expression and proliferation dynamics in mouse embryonic stem cells

**DOI:** 10.1038/s41540-017-0020-5

**Published:** 2017-08-03

**Authors:** Simon Godwin, Daniel Ward, Elisa Pedone, Martin Homer, Alexander G. Fletcher, Lucia Marucci

**Affiliations:** 1 Department of Engineering Mathematics, University of Bristol, Bristol, BS8 1UB UK; 2 School of Cellular & Molecular Medicine, University of Bristol, Bristol, BS8 1TD UK; 3 School of Mathematics and Statistics, University of Sheffield, Sheffield, S3 7RH UK; 4 Bateson Centre, University of Sheffield, Sheffield, S10 2TN UK; 5 BrisSynBio, University of Bristol, Bristol, BS8 1TQ UK

## Abstract

During development, pluripotency is a transient state describing a cell’s ability to give rise to all three germ layers and germline. Recent studies have shown that, in vitro, pluripotency is highly dynamic: exogenous stimuli provided to cultures of mouse embryonic stem cells, isolated from pre-implantation blastocysts, significantly affect the spectrum of pluripotency. 2i/LIF, a recently defined serum-free medium, forces mouse embryonic stem cells into a ground-state of pluripotency, while serum/LIF cultures promote the co-existence of ground-like and primed-like mouse embryonic stem cell subpopulations. The latter heterogeneity correlates with temporal fluctuations of pluripotency markers, including the master regulator Nanog, in single cells. We propose a mathematical model of Nanog dynamics in both media, accounting for recent experimental data showing the persistence of a small Nanog Low subpopulation in ground-state pluripotency mouse embryonic stem cell cultures. The model integrates into the core pluripotency Gene Regulatory Network both inhibitors present in 2i/LIF (PD and Chiron), and feedback interactions with genes found to be differentially expressed in the two media. Our simulations and bifurcation analysis show that, in ground-state cultures, Nanog dynamics result from the combination of reduced noise in gene expression and the shift of the system towards a monostable, but still excitable, regulation. Experimental data and agent-based modelling simulations indicate that mouse embryonic stem cell proliferation dynamics vary in the two media, and cannot be reproduced by accounting only for Nanog-dependent cell-cycle regulation. We further demonstrate that both PD and Chiron play a key role in regulating heterogeneity in transcription factor expression and, ultimately, mouse embryonic stem cell fate decision.

## Modelling gene expression and proliferation dynamics of pluripotent stem cells

Mouse embryonic stem cells (mESCs) are pluripotent cells, having the potential to turn into most other cell types. A team led by Lucia Marucci at the University of Bristol, and involving collaborators from the University of Sheffield, developed mathematical models to describe temporal dynamics of pluripotency genes in mESCs under different culture conditions. The team shows that the combination of feedback loops in the underlying gene regulatory networks, noise, and culture conditions fine-tunes gene expression dynamics and, consequently, the fate of mESCs. Experimental and modelling results highlight the interplay between pluripotency gene dynamics and cellular proliferation. Understanding the dynamical mechanisms that influence the fate of mESCs could guide the optimisation of culture protocols in both pluripotency and differentiation.

## Introduction

Mouse embryonic stem cells (mESCs) are pluripotent cells, isolated from the inner cell mass, which can be indefinitely expanded and retain pluripotency, or be pushed into specific differentiated states by proper stimuli in vitro, contributing to all germ layers when injected into host embryos.^1^ In recent years, significant research effort has been put into defining optimal culture conditions for pluripotency maintenance, and identifying protocols for efficient differentiation of mESCs.

The standard mESC culture medium is serum/LIF; it contains serum factors and the cytokine leukaemia inhibitory factor (LIF). This medium has been reported to confer on mESCs a heterogeneous expression and temporal fluctuations of pluripotency factors and regulators, including Nanog, Rex1, Stella, Esrrb and β-catenin.^2–6^ Notably, many of the metastable mESC genes are, directly or indirectly, regulated by Nanog,^7^ a master regulator of pluripotency and development.^8^ Importantly, mosaic expression patterns in serum/LIF result in an inhomogeneous propensity to differentiate in mESC subpopulations. This phenotype is reversible however: cells expressing Nanog Low levels, which are more prone to differentiate, can in time switch Nanog on, and vice versa for Nanog High cells.^3^

Such results prompted the identification of new culture conditions able to reduce heterogeneity and allow a standardised pluripotency phenotype. Recently, a new culture medium (2i/LIF) has been proposed^9^; it is serum-free and contains the two chemical inhibitors PD0325901 (MEK inhibitor,^10^ hereafter named PD) and CHIR99021 (glycogen synthase kinase-3 (Gsk3) inhibitor,^11^ hereafter named Chiron). When cultured in 2i/LIF, mESC reporter cell lines commonly used to monitor expression of Nanog and its direct target Rex1 (TNGA, which carries a stable green fluorescent protein (GFP) in one of the Nanog alleles,^3^ and Rex1GFPd2, in which a destabilised GFP is inserted into the Rex1 locus,^12^ respectively) show an almost complete elimination of the Nanog Low and Rex1 Low subpopulations observed under serum/LIF cultures, suggesting that 2i/LIF enables ground state pluripotency in vitro.^13^

The observed abolition of Nanog heterogeneity in 2i/LIF has recently been challenged,^14, 15^ as well as the reliability of TNGA mESCs as a reporter cell line, due to differences between endogenous Nanog and GFP degradation rates.^16^ A more recently-engineered Nanog reporter mESC cell line (Nd mESC) carries, under the Nanog regulatory regions, a destabilised Venus protein (Venus-Nuclear-PEST) with mRNA and protein half-lives comparable to endogenous Nanog.^17^ Of note, while enabling a highly dynamic Nanog readout, Nd mESCs have both Nanog alleles intact. As compared to TNGA mESCs, Nanog is still bistable in serum/LIF Nd cells, although with a smaller difference between the two Nanog Low and Nanog High (named NL and NH hereafter) subpopulations. Also, sorted cells re-establish the original bimodal distribution on a shorter time-scale.^15, 17^ In ground-state cultures, a small NL subpopulation is still present in Nd mESCs; consistently, stochastic fluctuations between the two states can be observed within a single cell-cycle (CC), with the same amplitude (i.e., difference between maximum and minimum fluorescence levels measured in single-cell time-lapse experiments) observed in serum/LIF cultured cells.^15^

Diverse molecular mechanisms have been proposed to explain the divergent dynamics in the two media, including Nanog transcriptional bursting,^18^ epigenetic mechanisms^19^ and microRNA regulation.^20^ Alongside these mechanisms, feedbacks in Gene Regulatory Networks (GRNs) of core pluripotency and differentiation factors play a key role in determining transcriptional dynamics and decision-making of mESCs.^21^

Mathematical modelling can be a powerful tool, not only to formalise dynamics observed experimentally, but also to suggest ad hoc perturbations to influence fate decision in mESCs.^22^ Different mathematical formalisms have been proposed to describe mESC culture-dependent transcriptional dynamics (see ref. 22 for a review), focusing on Nanog regulations. Although based on different hypotheses about the origin of heterogeneity and fluctuations of pluripotency genes in serum/LIF cultures, all these models fail to predict the above-mentioned recent findings of the persistence of a NL subpopulation in 2i/LIF.

Furthermore, in the systems biology literature we are not aware of any mathematical attempt to explain and compare proliferation dynamics of mESCs in the two media. cell-cycle progression and stem cell commitment have been shown to be linked processes^23^: pluripotent mESCs display a unique and singular cell-cycle defined by a fast proliferation rate, long S/G2 phase and short G1 phase.^24^ Conversely, during differentiation, the G1 phase duration increases in mESCs, hESCs, mouse and human iPSCs.^25, 26^ It has thus been proposed that a long G1 phase plays a key role in differentiation, while a short G1 phase might be involved in actively sustaining the pluripotent state.^26^ On the other hand, accumulation of mESCs in the G1 phase by inhibition of Cdk2 reduces their proliferation but does not affect pluripotency.^27^ Agent-based models, that incorporate GRN dynamics into cellular agents, can aid in unravelling the coupling between gene expression and cellular phenotypes in both systems and synthetic biology,^28, 29^ and help to explain the link between mESC proliferation and pluripotency. The only existing attempt in this direction recapitulates Nanog heterogeneity and sorting experiments of mESCs cultured in serum/LIF, but the model does not analyse the 2i/LIF scenario.^30^

Here, by mathematical modelling, we are able to recapitulate both the aforementioned persistence of a NL subpopulation in 2i/LIF, and mESC proliferation dynamics in both serum/LIF and 2i/LIF. We modelled the dynamics of an extended GRN, which includes genes differentially expressed in the two media, their transcriptional and posttranslational mutual interactions and the two inhibitors present in 2i/LIF. Numerical simulation and continuation results of our stochastic differential equation (SDE) model recapitulate existing data obtained with Nd mESCs, and indicate which of the network components are required to reproduce the experimental data. Agent-based simulations, calibrated to match mESC proliferation dynamics we measured experimentally, show that 2i/LIF cells possess a lower proliferation capacity than their serum/LIF counterpart, and that differences in Nanog levels alone cannot explain this phenotype, while Mycn qualifies as a regulator of cell-cycle progression. Overall, our results indicate that the complex interplay between feedback regulations, transcriptional noise, and the cell-cycle determines mESC dynamics and pluripotency, and that excitability might be an inherent feature of pluripotent cells.

## Results

### Network derivation: identification of transcriptional core interactions

We started from an existing GRN-based model,^31^ which formalised interactions among the core pluripotency transcription factors (Oct4, Sox2, Nanog and Rex1) to recapitulate mESC dynamics in serum/LIF and 2i/LIF media. The original network included Oct4-Sox2 heterodimer auto-activation, Oct4-Sox2 activation of Nanog, Nanog auto-activation, and the activation of Rex1 by both Nanog and the Oct4-Sox2 heterodimer. To account for medium-dependent differentiation signalling, an Fgf/Erk module was included, which inhibited Nanog’s auto-activation and was activated by Oct4. An additional phenomenological external differentiation signal, inhibiting the core pluripotency network and inhibited by Nanog, was also present.

With the aim of refining this model, and encompassing genes crucial for pluripotency maintenance and transcriptional heterogeneity, we used the workflow summarised in Supplementary Fig. S1. First, we re-analysed a published RNA-sequencing data set of mESCs cultured in serum/LIF and 2i/LIF,^13^ performing Gene Ontology (GO) analysis of the genes differentially expressed between the two media (3434 in total, fold change >2 and *p*-value <0.2) using the platform DAVID^32^ (Supplementary Table S1). We categorised genes by their associated biological processes (BPs), and retained only those whose BPs related to development and differentiation, proliferation, cell cycle, morphology or cell death (‘GO filter’, Supplementary Fig. S1). Genes differentially expressed in the two media and involved in the relevant BPs (2288 in total) were then filtered to include only those that interact with the core pluripotency factors (i.e., Oct4, Sox2 and Nanog). This was done using an updated compendium for mESC transcription factors, CODEX,^33, 34^ based on chromatin immunoprecipitation (ChIP) for transcription factors and histone modifications coupled to deep sequencing (ChIP-Seq), DNase-Seq and RNA-Seq data sets (Supplementary Fig. S1); genes regulating at least one of the core pluripotency factors were considered (34 genes, Supplementary Table S1). Rex1 present in the original network was removed, since it is only an output of the system (i.e., a marker of pluripotency) and does not directly regulate the pluripotent genes (thus, is not required to model the system dynamics).

From this analysis, we identified Mycn, Rest (RE1-silencing transcription factor) and Cdh7 (chromodomain helicase DNA binding protein 7) within the genes enriched in serum/LIF vs 2i/LIF, and Prdm14 (PR domain zinc finger 14) within the genes enriched in 2i/LIF. Of note, Rest, Mycn and Prdm14 had already been reported to significantly affect mESC pluripotency and the reprogramming of somatic cells.^35–37^ Chd7 was not included in the final network; although it targets gene enhancer elements and co-localises with ESC master regulation, it is not essential for self-renewal and pluripotency.^38^

To add gene interaction directions (activation or repression), we used the NIA mESC cell bank,^39^ generated by measuring the global expression patterns of mESCs upon perturbation of 54 transcription factors (Supplementary Fig. S1). Interactions between the core network genes Nanog, Oct4 and Sox2 were kept the same as in,^31^ where the authors considered the Oct4-Sox2 heterodimer complex,^40^ rather than the two single genes, regulating Nanog. Modelling the former regulation only has already been proven to enable good reproduction of mESC dynamics in serum/LIF^30, 31, 41–44^ and reduces the number of unknown parameters in the model. In the NIA bank analysis we found that Oct4 represses Nanog; consistently, in our network, the complex both activates Nanog,^40^ and also indirectly represses Nanog via the Fgf/Erk module (see below). The NIA bank analysis showed Nanog to be an auto-inhibitor, rather than an activator as in ref. 31. This interaction was kept as an auto-activator, again due to its demonstrated ability to provide a good reproduction of mESC dynamics; a comparison with the auto-inhibition case is provided below.

Since there were no data in the NIA bank for Rest and Prdm14, only interactions reported in the literature were included for the interaction of these genes with the rest of the network (see below). Of note, interactions found using CODEX but not confirmed in the NIA bank were removed (i.e., regulation of Mycn by Oct4 and Rest). In contrast, interactions found in the NIA bank but not present in CODEX were included (i.e., interaction between Mycn and Prdm14) as, although possibly being indirect interactions, they can affect the system dynamics. Regarding Mycn and Rest, in the NIA bank we found Oct4 and Sox2 to activate and inhibit both, respectively. Taken together as a heterodimer, with one activating (Oct4) and the other inhibiting (Sox2), there would be no resulting effect. Therefore no interaction from Oct4-Sox2 was included on Mycn and Rest.

### Medium-dependent signalling components

To account for medium-dependent Nanog dynamics, the original model^31^ considered the interaction of the MEK inhibitor (PD) with the core pluripotency network. However, the effects of the Gks3 inhibitor (Chiron) present in the 2i/LIF medium, and the resulting β-catenin stabilisation, were ignored. As in ref. 31, we model PD as a signal that weakens the inhibitory effect of Fgf/Erk signalling on Nanog. Given experimental evidence that PD alone is unable to sustain mESC clonal propagation,^1, 12^ and the key role of the Wnt/β-catenin pathway in pluripotency and reprogramming of somatic cells,^5, 45^ we additionally explored whether and how Chiron affects mESC expression patterns. We used a recently published ChiP-seq data set^46^ to identify the interactions of β-catenin with the core pluripotency network and the additional genes identified (see above). Using the list of genes differentially expressed between Chiron (Wnt pathway activator through inhibition of Gsk3) and XAV (tankyrase inhibitor antagonising Wnt signalling through stabilisation of the Axin2 inhibitor) reported in^46^ (Supplementary Table S1, Supplementary Fig. S1), β-catenin was found to inhibit directly both Mycn and Rest. We also included, using evidence from the literature, non-transcriptional Wnt/β-catenin pathway interactions known to be fundamental for pluripotency homoeostasis: Wnt/β-catenin inhibition of Tcf3 and Tcf3 inhibition of Oct4-Sox2 regulation of Nanog.^12^ Additional protein interactions were added from the literature for the positive feedback loop between Nanog and Rest,^37^ and Prdm14 inhibition of Fgf/Erk signalling.^47^

**Fig. 1:**
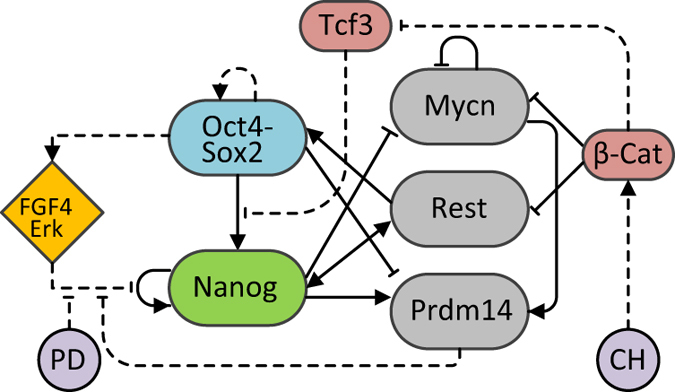
Scheme of the regulatory network. The core network is composed of the Oct4-Sox2 heterodimer and Nanog; Mycn, Rest and Prdm14 were added as genes differentially expressed in serum/LIF vs. 2i/LIF, and involved in feedback loops with the core network (detailed derivation in Supplementary Fig. S1). Fgf4/Erk, β-catenin and Tcf3 are added to account for the inputs (i.e., PD and Chiron -CH in the Figure-, the two inhibitors present in 2i/LIF). *Solid* and *dashed lines* indicate transcriptional and non-transcriptional interactions, respectively

In summary, the complete network represented in Fig. 1 is composed as follows:

- the heterodimer Oct4-Sox2 represses Prdm14 and activates both itself and Nanog, with the latter activation inhibited by Tcf3;
- Nanog activates itself, Rest, and Prdm14, and inhibits Mycn;
- Mycn inhibits itself and activates Prdm14;
- Rest activates Oct4-Sox2 and Nanog;
- Ffg4/Erk is activated by Oct4-Sox2 and represses Nanog auto-regulation; the latter regulation is inhibited by both PD and Prdm14;
- β-catenin is activated by Chiron (for simplicity, we merged Chiron inhibition of Gsk3, and Gsk3 inhibition of β-catenin as a single interaction), and inhibits Rest, Mycn and Tcf3.

### Dynamics in serum/LIF: Nanog bistability and noise-induced fluctuations

The SDE model represents activation/inhibition regulations in the network using Hill kinetics to encompass saturation with zero-mean Gaussian noise, as in ref. 31 to represent molecular background noise. For further details about model derivation, parameter settings and simulations, and full equations of the system, please refer to the Material and Methods section and Supplementary Information.

**Fig. 2:**
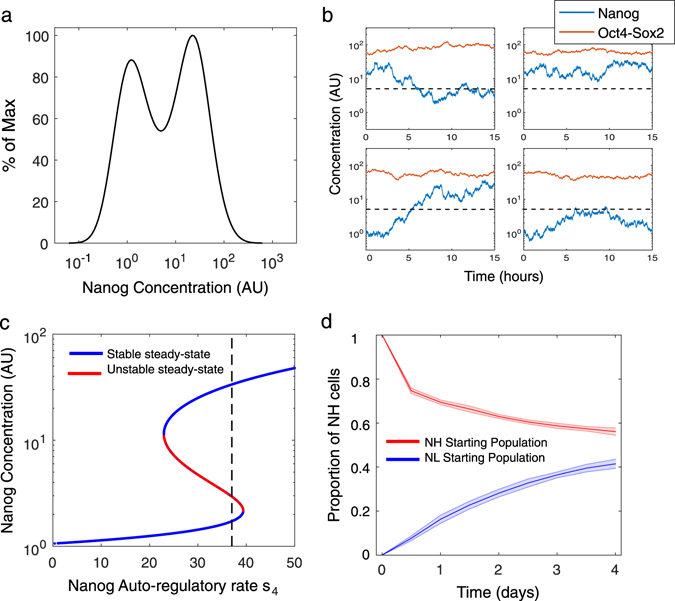
System dynamics and stability in serum/LIF (PD = Chiron = 0 AU) **a** Steady-state distribution of Nanog simulated in 1000 cells showing that approximately 50% of the cells are in Nanog High (NH) and 50% Nanog Low (NL) states. **b** Four representative time-course simulations of Nanog (*blue line*) and Oct4-Sox2 (*red line*) in single cells over 15 h. Oct4-Sox2 dimer concentration remains fairly constant compared to Nanog. The *horizontal black dotted line* represents the threshold between NH and NL. **c** Continuation of Nanog steady-state for the deterministic system (no noise). *Blue lines* are stable steady-states, while the *red line* is unstable. The maximal transcription rate, *s*_4_ (*dotted black line*), intersects both NH and NL steady-states, indicating bistability. **d** Sorting simulation: the dynamics of NH or NL cells (1000 for each state) are simulated over 4 days, and the proportion of cells in the NH state is recorded every 12 h (*n* = 10, *shaded areas* indicate standard deviation). The simulations show the cells tending towards the steady-state distribution of approximately 50% NH and 50% NL

We performed 50 simulations, each for 1000 individual cells (as independent realisations of the SDE model), to estimate the model parameters that consistently describe flow cytometry [fluorescence-activated cell sorting (FACS)] data of Nd mESCs cultured in serum/LIF.^15, 17^ The concentration of Nanog was recorded for each cell and distributions were captured at steady-state. Figure 2a shows a representative simulated distribution with a roughly equal number of cells in the NH and NL steady-states (the NH cells being 49.6 ± 1.5% of the overall population), correctly reproducing the bimodal distribution of Nanog observed experimentally in Nd cells (56.2 ± 8.0% of NH cells^15, 17^). Of note, the model can reproduce the fact that the Nd cells FACS distribution is narrower than the one measured in TNGA mESC experimental data,^48^ due to the higher stability of the reporter protein in the latter cells. In single-cell simulations, stochastic fluctuations of Nanog are observed between the Low and High state, in contrast to monostable dynamics of Oct4-Sox2 (Fig. 2b). The time-scale of our simulations matches experimental single-cell measurements of live Nd mESCs obtained in time-lapse experiments, which show fluctuations between the NH and NL states within a single cell-cycle (circa 12 h) (Fig. S2 in ref. 15), thus on a much shorter time-scale than the TNGA cells.^48^

We performed numerical bifurcation analysis (i.e., a numerical study of the changes in the dynamics and stability of a system upon variations in its parameters^49, 50^) continuing the Nanog steady-state as a function of the maximal auto-regulative transcription rate *s*_4_ (bifurcation parameter). A typical multistationary bifurcation plot is observed (Fig. 2c): within a certain *s*_4_ region, two stable steady-states (*blue lines*, corresponding to NL and NH states) co-exist, separated from the unstable steady-state (red line) by two saddle-node bifurcations. This result is in line with the hypothesis that the Nanog multimodal distribution in serum/LIF arises from its auto-regulatory feedback loops.^41, 43, 31^

Finally, we sought to reproduce sorting experiments, in which NL and NH Nd mESCs were FACS sorted, separately plated in serum/LIF medium and analysed for Nanog reporter expression over a 4-day time-course. Our simulations correctly match the experimental observation that both subpopulations can restore the original distribution within 4 days of culture (compare Fig. 2d here and experimental results in Fig. 3b in ref. 17).

**Fig. 3:**
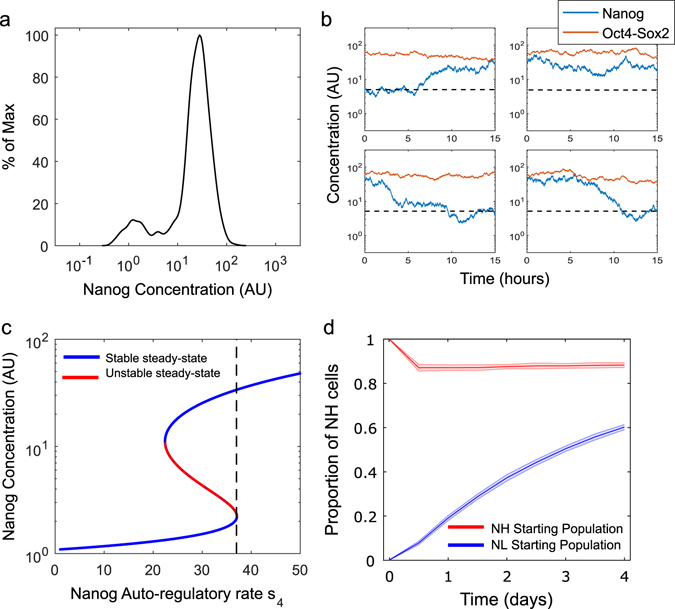
System dynamics and stability in 2i/LIF (PD = Chiron = 2 AU, Nanog transcriptional noise reduced by 20%) **a** Steady-state distribution of Nanog simulated in 1000 cells, showing that approximately 90% and 10% of the cells are in the NH and NL states, respectively. **b** Four representative time-course simulations of Nanog (*blue line*) and Oct4-Sox2 (*red line*) in single cells over 15 h. Similarly to the serum/LIF case, Oct4-Sox2 dimer concentration remains fairly constant compared to Nanog. The horizontal *black dotted line* represents the threshold between NH and NL. **c** Continuation of Nanog steady-state for the deterministic system (no noise). *Blue lines* are stable steady-states, while the *red line* is unstable. The maximal transcription rate, *s*_4_ (*dotted black line*), intersects both NH and NL steady-states, though is very close to the saddle-node bifurcation. **d** Sorting simulation: 1000 cells in the NH or NL state are simulated over 4 days, and the proportion of cells in the NH state is recorded every 12 h (*n* = 10, shaded areas indicate standard deviation). Cells restore the steady-state distribution of approximately 90% NH and 10% NL

### Dynamics in 2i/LIF: persistence of noise-induced fluctuations

As described in the Introduction, Nd mESCs contain a persistent small subpopulation of NL cells even in ground-state cultures (2i/LIF, experimental FACS distributions in Fig. 4b in ref. 17 and Fig. 5a in ref. 15), in contrast to previous evidence of a complete elimination of the NL subpopulation using a stable GFP reporter for Nanog.^9, 13, 51^ Existing mathematical formalisms, at least for parameters previously considered in the literature, predict a complete elimination of Nanog heterogeneity and temporal fluctuations in 2i/LIF cultures due to PD reducing Erk’s inhibition of Nanog.^31, 41^

**Fig. 4:**
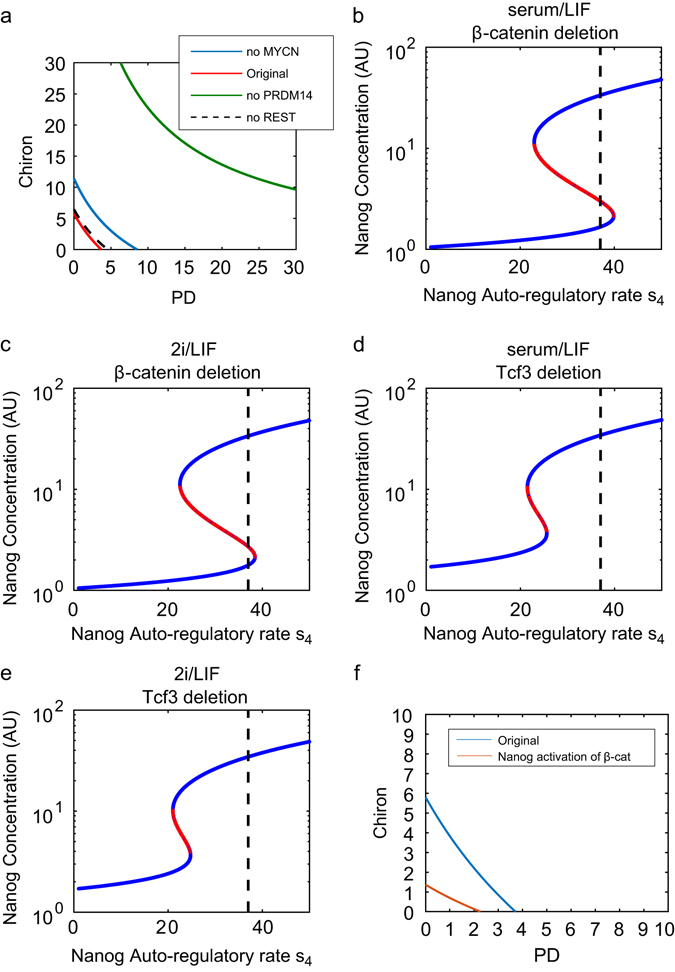
Bifurcation analysis of the full system, and upon single network factor deletion **a** Two-parameter continuation (PD and Chiron). The area above each curve denotes the parameter (i.e., PD and Chiron) region in which the deterministic system exhibits monostability (NH only) for the full network (*red line*), and upon single deletion of Rest (*dashed line*), Mycn (*blue line*), and PRDM14 (*green line*). The graph shows that both PD and Chiron, with varying levels depending on gene deletion, are required to enter the monostable region. **b**–**e** Continuation of Nanog steady-state for the deterministic system, varying maximal transcription rate *s*_4_, upon β-catenin deletion in serum/LIF (**b**) and 2i/LIF (**c**), and upon Tcf3 deletion in serum/LIF (**d**) and 2i/LIF (**e**). *Blue lines* are stable steady-states, while the *red lines* are unstable. **f** Two-parameter continuation (PD and Chiron). The area above the curve denotes the values of PD and Chiron in which the deterministic system exhibits monostability (NH only) for the full network, including an activation of Nanog on β-catenin (*red line*) comparing to the original full network with no additional activation of β-catenin (*blue line*)

**Fig. 5:**
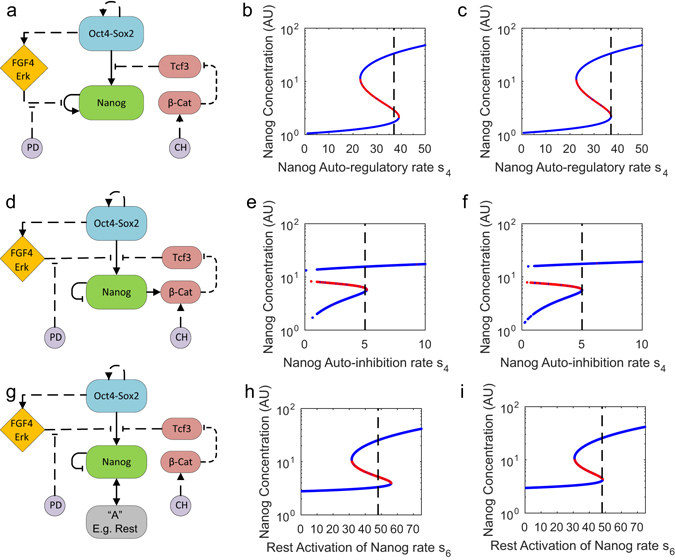
Simpler network models demonstrating bistability of Nanog. **a** Scheme of simplified network, composed of core factors Nanog, Oct4, Sox2; Fgf4/Erk, β-catenin and Tcf3 are added to account for the inputs (i.e., PD and Chiron (CH), the two inhibitors present in 2i/LIF). *Solid* and *dashed lines* indicate transcriptional and non-transcriptional interactions, respectively. **b**, **c** Continuation of Nanog steady-state for the deterministic system (no noise) in serum/LIF (**b**) and 2i/LIF (**c**). *Blue lines* are stable steady states, while *red lines* are unstable. The maximal transcription rate, *s*_4_ (*dotted black line*), intersects both NH and NL steady-states, indicating bistability in serum/LIF (**b**), but is close to the saddle-node bifurcation in 2i/LIF (**c**). **d** Scheme of Nanog auto-inhibitory simplified network, with additional activation of β-catenin by Nanog. **e**, **f** Continuation of Nanog steady-state for the deterministic system (no noise) in serum/LIF (**e**) and 2i/LIF (**f**). *Blue lines* are stable steady-states, while *red lines* are unstable. The maximal transcription rate of Nanog auto-inhibition, *s*_4_ (*dotted black line*), intersects both NH and NL steady-states, indicating bistability in serum/LIF (**e**), while being closer to the saddle-node bifurcation in 2i/LIF (**f**). **g** Scheme of Nanog auto-inhibitory simplified network, with additional positive feedback supplied via an extra factor, i.e. Rest. **h**, **i** Continuation of Nanog steady-state for the deterministic system (no noise) in serum/LIF (**h**) and 2i/LIF (**i**). *Blue lines* are stable steady-states, while *red lines* are unstable. The maximal transcription rate of Rest activation on Nanog, s_6_ (*dotted black line*), intersects both NH and NL steady-states, indicating bistability, in serum/LIF (**h**), and is very close to the saddle-node bifurcation in 2i/LIF (**i**)

To reproduce the system dynamics in 2i/LIF, we reduced the standard deviation of Nanog transcriptional noise by 20% as compared to its value in serum/LIF, consistent with experimental measurements of the number of mRNA molecules per cell obtained by single-molecule fluorescent in situ hybridisation in Nd mESCs.^15^ The model shows that 88.46 ± 1% of the cells are in the NH steady-state (Fig. 3a), matching experimental data (91.1 ± 3.1% in NH state^15, 17^).

Consistently, single-cell simulations over 15 h show fluctuations (Fig. 3b), in agreement with Nd single-cell time-lapses in ground-state cultures reported in ref. 15. Of note, for the specific levels of Chiron and PD used (Supplementary Information), the system is very close to the saddle-node bifurcation point (Fig. 3c). Figure 3d shows simulation results of the sorting experiment, with isolated NL and NH subpopulations approaching the original steady-state distribution after 4 days of culture.

To understand how the reduction of noise affects the system dynamics and stability, we sought to reproduce the same data keeping the Nanog noise term the same as in serum/LIF simulations (Supplementary Information). Obtaining a Nanog steady-state distribution and temporal dynamics equivalent to the previous reduced-noise scenario (giving now 87.27 ± 0.99% of NH cells, compare Figs. 3a, b with Supplementary Figure S2a and S2b, respectively) required an increase of the inputs PD and Chiron (Supplementary Information). With these parameters, the continuation of the Nanog steady-state against its auto-transcription rate *s*_4_ shows that the deterministic system is monostable (see the *black dotted line* only intersecting the NH steady-state in Supplementary Fig. S2c). Although the deterministic system has only NH as a stable attractor, due to the Erk signalling block, the transcriptional noise of Nanog causes about 10% of cells to enter NL. Supplementary Figure S2d shows a sorting simulation, where populations starting from purely NL and NH populations were simulated over 4 days. The cells tend towards the steady-state distribution seen in Supplementary Fig. 2a. However, when compared to simulations in Fig. 3d, the rate at which NL cells tend towards the steady-state is much faster. Sorting experiments in 2i/LIF cultures have not been performed, but would inform about the suitability of one approach vs. the other.

Taken together, our results show that, accounting for reduced noise in Nanog expression in ground-state cultures, 2i/LIF shifts the system towards the monostable regime while keeping it excitable.

### Dependence of the system stability on the GRN interactions

To understand how the shift of the system towards a monostable Nanog regime depends on the two inhibitors present in 2i/LIF and on the GRN topology, two-parameter continuations were performed on the variables PD and Chiron for the original network, and under single gene deletion (Fig. 4a). The area above each curve denotes the set of values of PD and Chiron for which the system is predicted to be monostable (NH state only).

**Fig. 6:**
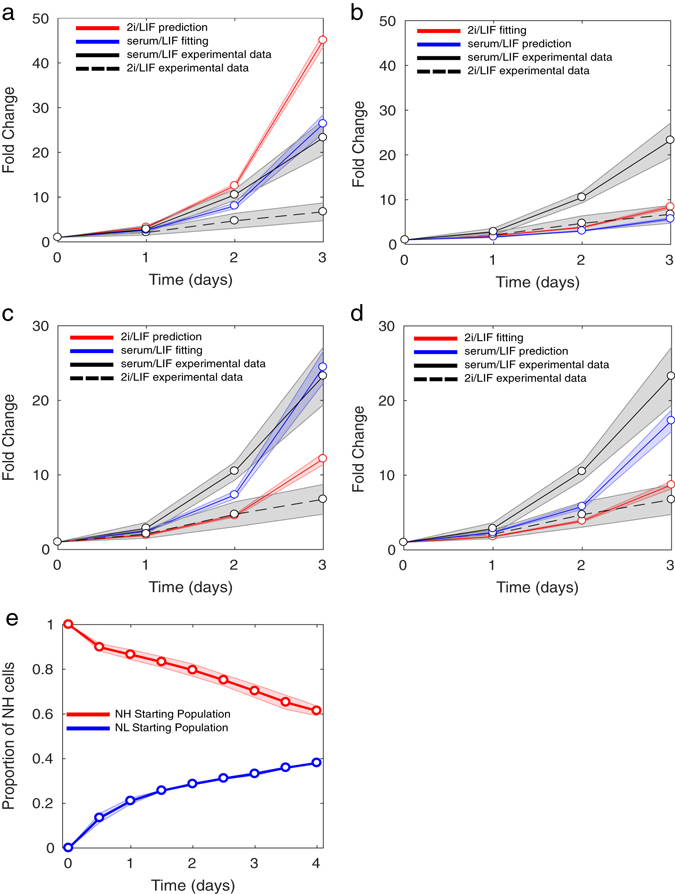
Agent-based modelling results for Nanog-dependent or Mycn-dependent cell-cycle (**a**, **b**) Nanog-dependent cell-cycle model simulations. **a** The *blue line* depicts the Nanog-dependent cell-cycle model fitted to serum/LIF proliferation data (data shown as *solid black line*, RMSE 2.35), while the *red line* is the predicted cell-growth in 2i/LIF (RMSE 22.59 as compared to 2i/LIF data, shown as *dashed black line*). Each cell cycle was independently sampled from a normal distribution with mean 8.5 h for NH and 13.5 h for NL, and standard deviation 1 h for both NH and NL. **b** The *red line* shows fitting of the Nanog-dependent cell-cycle model to 2i/LIF data (RMSE 1.07), with prediction of growth in serum/LIF plotted in *blue* (RMSE 11.01); cell cycles were allocated with mean 13.175 h for NH and 20.925 h for NL. **c**, **d** Mycn-dependent cell-cycle model simulations. **c** The *blue line* depicts the Mycn-dependent cell-cycle model fitted to serum/LIF data (*solid black line*, RMSE 2.02), while the *red line* is the predicted cell growth in 2i/LIF (data shown as *dashed black line*, RMSE 3.08). In this case the cell cycles were allocated from a normal distribution with mean 8.5 h for Mycn High (MH) and 15 h for Mycn Low (ML). **d** The *red line* shows the Mycn-dependent cell-cycle model fitted to 2i/LIF data (RMSE 1.33) with serum/LIF predicted growth dynamics plotted in blue (RMSE 4.41). The cell cycles were allocated with means 9.5 h for MH and 16 h for ML. (**e**) Agent-based simulations of Nanog sorting experiment (Mycn-dependent cell-cycle model) in serum/LIF. The *red* and *blue lines* depict the changing ratio of NH cells in the population starting from NH or NL state, respectively. In **a**–**d**, data are the average of four independent experiments. Cell count, performed every 24 h for 3 days, is plotted as fold change vs. count at day 0; Standard Error (SE) is shown as a *shaded grey region*. In **a**–**e**, all model simulations are the average of 15 independent simulations, with the *shaded regions* showing SE

The two-parameter continuation of the Nanog steady-state in the original network (no genes deleted) shows that the presence of both inhibitors is needed to leave the bistability region (Fig. 4a, *red line*). In particular, PD inhibits the auto-regulatory feedback of Nanog on itself, responsible for bistability, and Chiron strengthens Oct4-Sox2 activation of Nanog, facilitating the shift to the NH state.

The model predicts that Rest deletion (Fig. 4a, *black dotted line*) does not significantly impact system stability, while in the case of Mycn removal, monostability can be obtained only if PD and Chiron are increased (Fig. 4a, *blue line*); this is a consequence of the indirect activation of Nanog by Mycn through Prdm14 (Fig. 1). Indeed, the same trend is observed upon simulated Prdm14 deletion, but in this case a significant increase of inhibitors would be required (Fig. 4a, *green line*). Experimentally, Prdm14−/− mESCs cultured in 2i/LIF show heterogeneous Nanog expression, and defective differentiation potential.^35^ This confirms our results about the key role of Prdm14 for the system dynamics and consequent pluripotency phenotype. Simulating our SDE model for the Prdm14 deletion scenario, we found, at steady-state, a much greater proportion of cells expressing NL in serum/LIF (Supplementary Fig. S3a) and persistence of Nanog heterogeneity in 2i/LIF (Supplementary Fig. S3b), matching the mentioned experimental data in Prdm14−/− mESCs.

To understand the effect of β-catenin and Tcf3 on the stability of the deterministic system, continuations of the Nanog steady-state against its auto-transcription rate were performed upon β-catenin deletion, and Tcf3 deletion. Continuation upon β-catenin deletion for serum/LIF parameters (Fig. 4b) shows that the system shifts slightly further into the bistable regime, as indicated by the *dashed black line*, though the change is subtle (compare with Fig. 2c). A similar result is seen for 2i/LIF parameters as seen in Fig. 4c; here the system is shifted further into the bistable area (compare with Fig. 3c). More pronounced results are seen for Tcf3 deletion: using serum/LIF parameters (Fig. 4d), the system has moved entirely into the NH, monostable regime (compare with Fig. 2c). The same shift is seen, though even further, for 2i/LIF parameters as seen in Fig. 4e (compare again with Fig. 3c). These results are in line with experimental evidence that Tcf3 inhibition is associated with a significant delay in mESC differentiation.^52^ Note that bifurcation analysis was performed on the system without noise. As shown in the previous section (and also in ref. 53), noise can be sufficient to cause bimodality and temporal fluctuations in a positive feedback loop, even in a monostable regime.

### Nanog activation of β-catenin induces its stochastic fluctuations, but does not affect the overall system dynamics

Recently, we reported an indirect activation of Nanog on β-catenin (i.e., Nanog repression of β-catenin inhibitor Dkk1), which has important consequences for the successful reprogramming of mouse embryonic fibroblast to induced pluripotent stem cells.^5^

We analysed the dynamics of the system adding this extra interaction (for simplicity, we assumed that Nanog directly activates β-catenin; as the extra interaction with Dkk1 would eventually add some delay to the system). Simulating the system to reproduce serum/LIF cultures (i.e., using the same parameter set as in Fig. 2), the new interaction introduces a bimodal distribution at steady-state also for β-catenin (compare Supplementary Fig. S4b with Supplementary Fig. S4d). Thus, we successfully fitted the distribution observed in the β-catenin tagged EL55 mESC cell line and the difference between the β-catenin Low and High states reported in ref. 5. Nanog distribution in serum/LIF is almost unchanged after adding the new interaction (compare Supplementary Fig. S4a, S4c). Simulating the system in 2i/LIF culture conditions (noise and inputs as in Fig. 3), the Nanog distribution is again unaffected (compare Supplementary Fig. S4e, S4g), but cells are pushed toward a β-catenin High state (compare Supplementary Fig. S4f, S4h), with a small population of β-catenin-Low cells, qualitatively reproducing data in ref. 5. Interestingly, Nanog activation of β-catenin changes the dependency of the system on PD and Chiron, reducing the amount of inducers required to push the system towards a monostable regime (Fig. 4f). These results suggest that Nanog regulation of β-catenin does not affect the overall system stability dynamics, though it can sustain the shift from a bistable to a monostable regime.

### Reduced and modified network topologies can reproduce Nanog dynamics in the presence of at least one positive feedback

As described above, Nanog noise-induced temporal fluctuations arise in our model from the presence of a positive feedback loop in the network topology (i.e., Nanog auto-regulation, Fig. 1). We investigated whether reduced networks, which still keep such a loop, could recapitulate Nanog dynamics in both media.

First, we analysed the dynamics of a reduced network comprising the core factors Nanog, Oct4, Sox2 with an Fgf/Erk module only, the Wnt/β-catenin genes β-catenin and Tcf3 and the two inhibitors PD and Chiron (Fig. 5a). Interactions kept from the full network have the same parameter values; only interactions that were not directly comparable (i.e., those involving Fgf/Erk, PD and Prdm14) were altered to match experimental data (Supplementary Information). Results of continuation of the Nanog steady-state against its auto-regulatory rate are comparable to those obtained with the full network under the same parameters for inputs and noise (compare Fig. 5b, c with Figs. 2c and 3c for serum/LIF and 2i/LIF, respectively). These results confirm that Nanog auto-activation is sufficient to explain bistability of the system.

As in our NIA bank analysis, two independent studies have recently reported that Nanog might function as an auto-inhibitor of itself rather than an activator.^54, 55^ We investigated if we could still predict bistability considering auto-inhibition instead of auto-activation. Starting from the reduced model in Fig. 5a, we further added Nanog activation of β-catenin, which results in an indirect positive feedback loop acting on Nanog through β-catenin and Tcf3 (Fig. 5d). Changing the original parameters in such a way that this indirect positive feedback loop is strong enough (i.e., by increasing the system’s non-linearity via Hill coefficient values and sensitivity to β-catenin via Michaelis–Menten constants) we obtained a bistable regime in serum/LIF and a shift of the system towards the monostable regime in 2i/LIF (Fig. 5e, f). Similarly, we reproduced the system dynamics neglecting Nanog activation of β-catenin, but instead including another positive feedback loop present in the full network (i.e., formed by the reciprocal interaction between Nanog and Rest, Fig. 5g; see continuations of Nanog steady-state against Rest activation rate in serum/LIF and 2i/LIF in Fig. 5h, i, respectively). The interaction with Rest was slightly altered in the reduced network, compared to the full network, to increase its nonlinearity (i.e., increasing Hill coefficient values, Supplementary Information). Taken together, these results indicate that if Nanog auto-activation is absent other positive feedback loops in the full network can still recapitulate bistability.

Another interaction present in the full network, which is debated in the literature, is β-catenin’s inhibition of Mycn (predicted in the data set we used,^46^ although other reports suggest the existence of a positive feedback between Mycn and β-catenin^56^). We performed simulations for two different scenarios: no interaction between Mycn and β-catenin, and β-catenin activation of Mycn (Supplementary Figs. S5 and S6, respectively). Comparing the dynamics using these topologies (Supplementary Figs. S5b–S5e and S6b–S6e) with those of the original network (Figs. 2c, 2a, 3c, 3a and 4a) we found that β-catenin’s inhibition of Mycn is not necessary to reproduce Nanog dynamics in the two media.

### Agent-based analysis of the system dynamics

We developed an agent-based version of the model, accounting for cell division (shown schematically in Supplementary Fig. S7a) in order to reproduce not only Nanog dynamics, but also the progression of mESCs through the cell cycle in the two media. The few reports that compared experimentally the progression of the G1 phase in metastable vs. ground-state mESC cultures disagree in their conclusions. One recent study showed that the G1-phase of mESCs grown in 2i/LIF is shorter than in serum/LIF.^57^ This is in contrast to measurements of a slower cell cycle in 2i/LIF due to a longer G1 phase reported in ref. 58, and comparable proliferation rates in the two media reported in an independent study.^59^ We therefore performed 3-day proliferation assays of wild-type mESCs cultured in serum/LIF or 2i/LIF (Materials and Methods); as shown in Fig. 6a–d, ground-state cultured mESCs show a significantly slower proliferation rate, as compared to serum/LIF cultured cells.

In order to reproduce our experimental data in agent-based simulations, we used the serum/LIF data for fitting and the 2i/LIF data for validation, and vice versa (Supplementary Information). We first coupled cell-cycle (CC) progression to the expression of Nanog, given recent experimental and modelling results in ref. 30, which suggest that at least in serum/LIF, Nanog expression levels correlate positively with mESC proliferation rate. By allocating different CC times to NH and NL cells (mean CC_NH_ = 8.5 h and CC_NL_ = 13.5 h, and standard deviations of 1 h), we could match the serum/LIF experimental proliferation data, with a root-mean squared error (RMSE) between the data and model of 2.35 (Fig. 6a). Furthermore, simulations showed a distribution of Nanog of approximately 50% NH and 50% NL cells (Supplementary Fig. S7b), consistent with the SDE model (Fig. 2) and experimental data.^15, 17^ Using the parameters fitted to the serum/LIF data to predict cell growth in 2i/LIF resulted in an increase in the NH subpopulation, as observed in the SDE model (Supplementary Fig. S7c); however, there was a large discrepancy between the proliferation profile and the recorded experimental values, with a RMSE of 22.59 (approx. 10× the error in the serum fitting, Fig. 6a).

We next used the 2i/LIF data for fitting, and the serum/LIF data for validation: by increasing the Nanog-dependent CC duration in both NH and NL populations by 55% (CC_NH_ = 13.175 h and CC_NL_ = 20.925 h) we were able to reproduce 2i/LIF proliferation dynamics, with a RMSE of 1.07 (Fig. 6b). However, fitted parameters performed poorly at predicting serum/LIF dynamics (RMSE 11.01, approx. 4× the error in our serum fitting, Fig. 6b). These results indicate that the previously suggested Nanog-dependent only model for CC progression^30^ is unable to reproduce both the extensively reported increase in NH cells^1, 5, 9, 12, 60^ and a reduction in proliferation in ground state cultures we measured here.

We therefore sought another possible candidate gene in the underlying GRN (Fig. 1) whose expression might be responsible for the different proliferation dynamics observed in the two media. The Myc family of transcription factors has been reported to positively regulate cell growth and proliferation in various cell types,^61^ and is a master regulator of mESC proliferation.^62^

Therefore, we adapted the model by allocating the CC duration based on whether the mother of a newly divided cell is in the Mycn High (MH) or Mycn Low (ML) state. Of note, in our GRN, Mycn is inhibited by Nanog, and, therefore, also exhibits a bimodal distribution in serum/LIF, with reduced levels in 2i/LIF, consistent with experimental observations.^13^ In a similar way to the Nanog-dependent CC model, we first fitted the serum/LIF data set, obtaining a RMSE of 2.02 (Fig. 6c, 14% improvement on the Nanog model) with CC_MH_ = 8.5 h and CC_ML_ = 15 h and a distribution of Nanog of approximately 50% NH and 50% NL cells (Supplementary Fig. S7d). Importantly, using these values to predict the 2i/LIF data set gave an 86% improvement on the Nanog model, with a RMSE of 3.08 (Fig. 6c) and Nanog distribution comparable to the SDE model (Supplementary Fig. S7e). When fitting 2i/LIF proliferation data, the Mycn model not only gave good fitting results (CC_MH_ = 9.5 h and CC_ML_ = 16 h, RMSE 1.33, Fig. 6d), but also performed acceptably in predicting dynamics in serum/LIF (RMSE 4.41, Fig. 6d). When fitting the two data sets independently, we found a difference in CC duration of 8%, as opposed to the 55% needed to adapt the Nanog model to the 2i/LIF data set. Finally, we further validated the agent-based model (using parameters as in Fig. 6c) performing NL and NH sorting simulations in serum/LIF (Fig. 6e): coupling Mycn to the cell cycle also reproduces experimental data,^17^ with restoration of the original distribution within 4 days, as seen in our SDE model (Fig. 2d).

While the fitting results using a Mycn-dependent CC model were a significant improvement on the Nanog dependent model, the predictions were still not perfect (Figs. 6c and d); these discrepancies might be due to the simplified CC model we are using (in which daughter cells inherit the protein concentrations from the mother cell, and maintain a fixed cell cycle time until next division, Supplementary Information and ref. 30). Also, it may be noted that we are focusing on a limited number of genes that are differentially expressed between the two cultures conditions; other cell cycle regulators, not included here as not relevant to determine Nanog dynamics, might also affect the different proliferation kinetics that we measured.

## Discussion

Understanding the causes and consequences of heterogeneity in pluripotent stem cells is crucial to define optimal protocols for pluripotency maintenance and robust differentiation.

In this paper we focused on pluripotency-related factor dynamics of an isogenic mESC population, including feedback regulations, external stimuli provided in culture media, and coupling to the CC.

Our computational results show that heterogeneous expression and fluctuations of pluripotency genes in serum/LIF cultures arise as noise-induced bistable dynamics from the combination of positive feedback loops and transcriptional noise, in line with previous computational studies.^31, 41, 43, 60^ Recent experimental results^63^ based on mESC single-cell resolution data (single-molecule RNA-FISH and quantitative time-lapse imaging) are incompatible with alternative models, which suggest oscillatory^43^ or excitable^48^ dynamics in serum/LIF. In our model, bistability is tightly regulated not only by Fgf, known to destabilise the pluripotent state,^64^ but also by the canonical Wnt pathway, demonstrated experimentally to avoid differentiation of mESCs into epiblast stem cells.^65^ Indeed, our numerical continuation shows that the contribution of both PD and Chiron regulates the stability and dynamics of the system, and that deletion of Tcf3 causes a shift into the Nanog High state.

Our model suggests that the ground-state of pluripotency is excitable, i.e., a subpopulation of cells can still leave the Nanog High state. Our results re-conciliate experimental evidence of the overall homogeneity of pluripotency factors^9, 13, 51, 63^ and the co-existence of subpopulations with different fate potential in 2i/LIF,^14, 66^ and correctly predict the effect of gene knock-outs on the overall stability of the pluripotent state. It can be argued that other parameter settings might have been able to reproduce the experimental data considered here, given that we did not measure them directly. Hill kinetics are used in all the models we developed, with consequent assumptions on the system (e.g., the cell is well stirred, and concentrations and rate constants are not spatially dependent^67^). However, our qualitative analysis still provides useful insights into the system stability and dynamic behaviour. We showed that reduced or modified networks can reproduce Nanog dynamics, but each of the new genes introduced in our GRN has a key role in determining the system behaviour: Prdm14 interaction with Fgf/Erk maintains the right proportion of NH to NL cells; Rest, through its interaction with Nanog, ensures the presence of a positive feedback loop (even if Nanog auto-repression is considered), and Mycn is needed for CC regulation. Indeed, it was not possible to reproduce the measured mESC growth kinetics in both media by accounting only for the coupling between Nanog and cell proliferation previously proposed.^30^ Our model and experimental data indicate, in agreement with recent evidence,^58^ that 2i/LIF-cultured mESCs show reduced proliferation as compared to serum/LIF conditions. In our view, the causal relationship between CC progression and ground-state pluripotency needs to be better characterised, as well as the extent to which G1 phase lengthening affects pluripotency marker homogeneity. Extensions of the agent-based models proposed here, informed by single-cell data and including additional genes differentially expressed between serum/LIF and 2i/LIF and involved in CC regulation, might be highly informative.

Our results indicate that the perturbation of pluripotency and CC regulators must be properly balanced for pluripotency maintenance, and could inspire further research for the definition of chemical media affecting both signalling pathways and cell proliferation.

Lineage-associated gene expression is still present in 2i/LIF conditions,^14^ reinforcing the biological hypothesis that there might still be functional heterogeneity related to extra-embryonic potential^1^; we also showed that multiple feedback loops can alter the dynamic equilibrium of ground-state pluripotency. Further investigation is needed to definitively assess if heterogeneous and fluctuating expression profiles might be detrimental for in vitro state pluripotency maintenance,^68^ or might contribute to unbiased cell fate determination in the exit from pluripotency.^7, 63^

## Materials and Methods

### Model derivation and parameterisation

We used in the SDE model Hill kinetics for transcriptional interactions to account for saturation; when a gene was regulated transcriptionally by more than one factor, Hill terms were summed. Regarding non-transcriptional interactions, Chiron was assumed to decrease the degradation of β-catenin, and β-catenin assumed to increase the degradation of Tcf3. Such regulations were modelled using Hill terms within the degradation terms of the respective equations. Tcf3 was assumed to decrease the binding rate of the Oct4-Sox2 heterodimer activation of Nanog. We used an mRNA steady-state assumption, modelling mRNA and protein dynamics in a single term, as in ref. 31. Noise was modelled with an additive zero-mean Gaussian term, where noise term parameters refer to the standard deviation of the normal distribution being randomly sampled from. Time was multiplied by a normalisation factor to match the time-scale of time-lapses in ref. 15. Parameters of the interactions kept from the reference GRN^31^ were maintained as close as possible to the values in the model proposed therein, and adapted to match, at steady-state, the fold change direction of genes in serum/LIF vs. 2i/LIF reported in ref. 13, and Nanog steady-state distributions in ref. 15. Degradation rates were fixed, and Hill coefficients for interactions not present in the original model^31^ were fixed at 1.

SDE simulations settings (including equations, parameter description and values), continuation analysis settings and details of the agent-based simulations are given in Supplementary Information.

### Cell proliferation experiments

Mouse ESCs (E4Tg2a and GS) were grown in Dulbecco’s modified Eagle’s medium supplemented with 20% foetal bovine serum (Sigma), 1× nonessential amino acids, 1× GlutaMax, 1 × 2-mercaptoethanol and 1000U/ml murine recombinant LIF (Peprotech) or in NDiff227 Neural differentiation medium (Stem Cells, SCS-SF-NB-02) supplemented with 1000U/ml murine recombinant LIF (Peprotech), 3 μM CHIRON-99021(Selleck) and 1 μM PD0325901(Selleck).

Mouse ESCs, grown in Serum/LIF or 2i/LIF for four passages, were seeded on gelatin-coated 24 wells/plates at the concentration of 10–20 ×1 0^3^ per well 24h before starting the experiment.

Every day, for 3 consecutive days, cells were tripsinized and 10µl of cell suspension were manually counted using a Neubauer chamber. Data are means ± Standard Error (*n* = 4, 2 replicates for each cell line).

### Data availability

Files needed to reproduce simulations in the paper are available in the Figshare repository (10.6084/m9.figshare.5053330).

## Electronic supplementary material

Supplemental Material

Supplemental Material
