# Supplementary material for: An extended model for culture-dependent heterogenous gene expression and proliferation dynamics in mouse embryonic stem cells
Source: NPJ Syst Biol Appl. 2017 Aug 3;3:19. doi: 10.1038/s41540-017-0020-5 (PMC5543144; doi:10.1038/s41540-017-0020-5)
Supplement: Supplementary file 2 — Supplemental Material [file 41540_2017_20_MOESM2_ESM.pdf]

SUPPLEMENTARY INFORMATION

AN EXTENDED MODEL FOR CULTURE-DEPENDENT HETEROGENOUS GENE EXPRESSION AND PROLIFERATION DYNAMICS IN MOUSE EMBRYONIC STEM CELLS

Simon Godwin, Daniel Ward, Elisa Pedone, Martin Homer, Alexander G. Fletcher, Lucia Marucci

Supplementary methods

Equations and parameter values

Interactions among genes in the network are described using Hill kinetics, to encompass saturation, as in (1). Activations are in the form:

$$\frac{S_i * [x]^n}{k^n + [x]^n}$$

while inhibitions are in the form:

$$\frac{S_i * k^n}{k^n + [x]^n}$$

S_i terms are the maximum transcription rates of the corresponding Hill terms; k_i terms are Michaelis-Menten constants, and represent the concentration of a substrate, corresponding to its Hill term, required for the transcription rate to reach half its maximum (i.e. $S_i/2$); d_i are degradation rates (first order kinetics); n is a scaling factor; $\phi(0,\sigma_i)$ terms represent added noise, sampled from a zero mean Gaussian; σ_i represents the noise amplitude. Where present, α_i represents basal activity. Note that for the networks in Figure 5, only continuation analysis of the systems without noise was performed; thus, the noise terms are not present in the corresponding equations. p is the strength of Fgf4/Erk signalling inhibition of Nanog, and is a function of both Prdm14 and PD. All simulation files are available upon request.

Equations of the full system (network in Figure 1)

$$p = \frac{s_{p1} * k_{p1}}{k_{p1} + [PR]} + \frac{s_{p2} * k_{p2}}{k_{p2} + [PD]}$$

$$\frac{d[OS]}{dt} = \mathrm{n} * \left(\frac{s_{1,2} * [OS]^2}{(k + [OS])^2 * dO * dS} + \frac{s_3 * [R]}{k + [R]} - dOS * [OS] \right) + \varphi(0, \sigma_{os}) * [OS]$$

$$\frac{d[N]}{dt} = \mathrm{n} * \left(\frac{s_4 * N^2}{k^2 + [N]^2 + p * [E]} + \frac{s_5 * [OS]}{k + [OS] + [Tcf3]} + \frac{s_6 * [R]}{k + [R]} - dN * [N] \right) + \varphi(0, \sigma_N) * [N]$$

$$\frac{d[M]}{dt} = \mathrm{n} * \left(\frac{s_7 * k}{k + [M]} + \frac{s_8 * k}{k + [N]} + \frac{s_9 * k_m}{k_m + [B]} - dM * [M] \right) + \varphi(0, \sigma_M) * [M]$$

$$\frac{d[PR]}{dt} = \mathrm{n} * \left(\frac{s_{10} * k}{k + [OS]} + \frac{s_{11} * [N]}{k + [N]} + \frac{s_{12} * [M]}{k + [M]} - dPR * [PR] \right) + \varphi(0, \sigma_{PR}) * [PR]$$

$$\frac{d[R]}{dt} = \mathrm{n} * \left(\frac{s_{13} * [N]}{k + [N]} + \frac{s_{14} * k_r}{k_r + [B]} - dR * [R] \right) + \varphi(0, \sigma_R) * [R]$$

$$\frac{d[E]}{dt} = \mathrm{n} * \left(\frac{s_{15} * [OS]}{k + [OS]} - dE * [E] \right)$$

$$\frac{d[B]}{dt} = \mathrm{n} * \left(\alpha_1 - dB * \left(\frac{s_b * k_b}{k_b + [CH]} \right) * [B] \right) + \varphi(0, \sigma_B) * [B]$$

$$\frac{d[Tcf3]}{dt} = \mathrm{n} * \left(\alpha_2 - dTc * \left(1 + \frac{s_t * [B]}{k_t + [B]} \right) * [Tcf3] \right) + \varphi(0, \sigma_T) * [Tcf3]$$

N=Nanog; *OS*=Oct4-Sox2; *M*=Mycn; *PR*=Prdm14; *R*=Rest; *E*=Fgf4/Erk; *B*=β-catenin; *Tcf3*=Tcf3.

Equations of the extended system (network in Figure 1, plus activation of Nanog on β-catenin).

$$p = \frac{s_{p1} * k_{p1}}{k_{p1} + [PR]} + \frac{s_{p2} * k_{p2}}{k_{p2} + [PD]}$$

$$\frac{d[OS]}{dt} = \mathrm{n} * \left(\frac{s_{1,2} * [OS]^2}{(k + [OS])^2 * dO * dS} + \frac{s_3 * [R]}{k + [R]} - dOS * [OS] \right) + \varphi(0, \sigma_{os}) * [OS]$$

$$\frac{d[N]}{dt} = \mathrm{n} * \left(\frac{s_4 * N^2}{k^2 + [N]^2 + p * [E]} + \frac{s_5 * [OS]}{k + [OS] + [Tcf3]} + \frac{s_6 * [R]}{k + [R]} - dN * [N] \right) + \varphi(0, \sigma_N) * [N]$$

$$\frac{d[M]}{dt} = \mathrm{n} * \left(\frac{s_7 * k}{k + [M]} + \frac{s_8 * k}{k + [N]} + \frac{s_9 * k_m}{k_m + [B]} - dM * [M] \right) + \varphi(0, \sigma_M) * [M]$$

$$\frac{d[PR]}{dt} = \mathrm{n} * \left(\frac{s_{10} * k}{k + [OS]} + \frac{s_{11} * [N]}{k + [N]} + \frac{s_{12} * [M]}{k + [M]} - dPR * [PR] \right) + \varphi(0, \sigma_{PR}) * [PR]$$

$$\frac{d[R]}{dt} = \mathrm{n} * \left(\frac{s_{13} * [N]}{k + [N]} + \frac{s_{14} * kr}{k_r + [B]} - dR * [R] \right) + \varphi(0, \sigma_R) * [R]$$

$$\frac{d[E]}{dt} = \mathrm{n} * \left(\frac{s_{15} * [OS]}{k + [OS]} - dE * [E] \right)$$

$$\frac{d[B]}{dt} = \mathrm{n} * \left(\alpha_1 + \frac{s_{nb} * [N]}{k + [N]} - dB * \left(\frac{s_b * k_b}{k_b + [CH]} \right) * [B] \right) + \varphi(0, \sigma_B) * [B]$$

$$\frac{d[Tcf3]}{dt} = \mathrm{n} * \left(\alpha_2 - dTc * \left(1 + \frac{s_t * [B]}{k_t + [B]} \right) * [Tcf3] \right) + \varphi(0, \sigma_T) * [Tcf3]$$

N=Nanog; *OS*=Oct4-Sox2; *M*=Mycn; *PR*=Prdm14; *R*=Rest; *E*=Fgf4/Erk; *B*=β-catenin; *Tcf3*=Tcf3.

Parameter values (network in Figure 1)

s_{p1}	30*	s_{14}	20*	dM	1#
s_{p2}	30*	s_{15}	2*	dPR	1#
k_{p1}	5	s_b	50*	dR	1#
k_{p2}	15	s_t	20*	dE	1#
$s_{1,2}$	75*	α_1	1*	dB	1#
s_3	1*	α_2	40*	dTc	1#
s_4	37*	k	10	σ_{OS}	0.02
s_5	2*			σ_N	0.05(0.04 for PD, Chiron=2)
s_6	0.01*	k_r	0.05	σ_M	0.02
s_7	10*	k_m	0.05	σ_{PR}	0.02
s_8	100*	k_t	10	σ_R	0.02
s_9	20*	k_b	1	σ_B	0.02
s_{10}	15*	dO	1#	σ_T	0.02
s_{11}	30*	dS	1#	s_{nb}	10*
s_{12}	10*	dOS	1#	n	0.005
s_{13}	0.1*	dN	1#		

(* molecules/minute; # 1/minute)

Equations of simplified network 1 (Figure 5a)

$$p = \frac{s_p * k_p}{k_p + [PD]}$$

$$\frac{d[OS]}{dt} = \text{n} * \left(\frac{s_{1,2} * [OS]^2}{(k + [OS])^2 * dO * dS} - dOS * [OS]\right)$$

$$\frac{d[N]}{dt} = \text{n} * \left(\frac{s_4 * N^2}{k^2 + [N]^2 + p * [E]} + \frac{s_3 * [OS]}{k + [OS] + [Tcf3]} - dN * [N]\right)$$

$$\frac{d[E]}{dt} = \text{n} * \left(\frac{s_5 * [OS]}{k + [OS]} - dE * [E]\right)$$

$$\frac{d[B]}{dt} = \text{n} * \left(\alpha_1 - dB * \left(\frac{s_b * k_b}{k_b + [CH]}\right) * [B]\right)$$

$$\frac{d[Tcf3]}{dt} = \text{n} * \left(\alpha_2 - dTc * \left(1 + \frac{s_t * [B]}{k_t + [B]}\right) * [Tcf3]\right)$$

N=Nanog; *OS*=Oct4-Sox2; *E*=Fgf4/Erk; *B*=β-catenin; *Tcf3*=Tcf3.

Parameter values (Network in Figure 5a)

**s_p**	35*	**dN**	1#
**k_p**	25	**dM**	1#
**$s_{1,2}$**	75*	**dPR**	1#
**s_3**	2*	**dR**	1#
**s_4**	37*	**dE**	1#
**s_5**	2*	**dB**	1#
**s_b**	50*	**dTc**	1#
**s_t**	20*	**n**	0.005
**α_1**	1*		
**α_2**	40*		
**k**	10		
**k_t**	10		
**k_b**	1		
**dO**	1#		
**dS**	1#		
**dOS**	1#		

(* molecules/minute; # 1/minute)

Equations of simplified network 2 (Network in Figure 5d)

$$p = \frac{s_p * k_p}{k_p + [PD]}$$

$$\frac{d[OS]}{dt} = \text{n} * \left(\frac{s_{1,2} * [OS]^2}{(k + [OS])^2 * dO * dS} - dOS * [OS] \right)$$

$$\frac{d[N]}{dt} = \text{n} * \left(\frac{s_4 * k}{k + [N]} + \frac{s_3 * [OS]}{k + [OS] + [Tcf3]^2 + p * [E]} - dN * [N] \right)$$

$$\frac{d[E]}{dt} = \text{n} * \left(\frac{s_5 * [OS]}{k + [OS]} - dE * [E] \right)$$

$$\frac{d[B]}{dt} = \text{n} * \left(\alpha_1 + \frac{S_{nb} * [N]^3}{k^3 + [N]^3} - dB * \left(\frac{s_b * k_b}{k_b + [CH]} \right) * [B] \right)$$

$$\frac{d[Tcf3]}{dt} = \text{n} * \left(\alpha_2 - dTc * \left(1 + \frac{s_t * [B]^3}{{k_t}^3 + [B]^3} \right) * [Tcf3] \right)$$

N=Nanog; *OS*=Oct4-Sox2; *E*=Fgf4/Erk; *B*=β-catenin; *Tcf3*=Tcf3.

Parameter values (Network in Figure 5d)

**s_p**	35*	**dN**	1#
**k_p**	5	**dM**	1#
**$s_{1,2}$**	75*	**dPR**	1#
**s_3**	35*	**dR**	1#
**s_4**	5*	**dE**	1#
**s_5**	2*	**dB**	1#
**s_b**	50*	**dTc**	1#
**s_t**	15*	**n**	0.005
**s_{nb}**	10*		
**α_1**	1*		
**α_2**	40*		
**k**	10		
**k_t**	0.15		
**k_b**	250		
**dO**	1#		
**dS**	1#		
**dOS**	1#		

(* molecules/minute; # 1/minute)

Equations of simplified network 3 (Network in Figure 5g)

$$p = \frac{s_p * k_p}{k_p + [PD]}$$

$$\frac{d[OS]}{dt} = \mathrm{n} * \left(\frac{s_{1,2} * [OS]^2}{(k + [OS])^2 * dO * dS} - dOS * [OS] \right)$$

$$\frac{d[N]}{dt} = \mathrm{n} * \left(\frac{s_4 * k}{k + [N]} + \frac{s_3 * [OS]}{k + [OS] + [Tcf3] + p * [E]} + \frac{s_6 * [R]^2}{{k_n}^2 + [R]^2} - dN * [N] \right)$$

$$\frac{d[R]}{dt} = \mathrm{n} * \left(\frac{s_7 * [N]^2}{k^2 + [N]^2} - dR * [R] \right)$$

$$\frac{d[E]}{dt} = \mathrm{n} * \left(\frac{s_5 * [OS]}{k + [OS]} - dE * [E] \right)$$

$$\frac{d[B]}{dt} = \mathrm{n} * \left(\alpha_1 - dB * \left(\frac{s_b * k_b}{k_b + [CH]} \right) * [B] \right)$$

$$\frac{d[Tcf3]}{dt} = \mathrm{n} * \left(\alpha_2 - dTc * \left(1 + \frac{s_t * [B]}{k_t + [B]} \right) * [Tcf3] \right)$$

N=Nanog; *OS*=Oct4-Sox2; *R*=Rest; *E*=Fgf4/Erk; *B*=β-catenin; *Tcf3*=Tcf3.

Parameter values (Network in Figure 5g)

s_p	35*	**dO**	1#
k_p	10	**dS**	1#
$s_{1,2}$	75*	**dOS**	1#
s_3	6*	**dN**	1#
s_4	1*	**dM**	1#
s_5	2*	**dPR**	1#
s_6	48	**dR**	1#
s_7	50	**dE**	1#
s_b	50*	**dB**	1#
s_t	20*	**dTc**	1#
α_1	1*	**n**	0.005
α_2	40*		
**k**	10		
k_n	45		
k_t	6		
k_b	1		

(* molecules/minute; # 1/minute)

Equations of the full system, with no regulation of β-catenin on Mycn (Network in Figure S5a)

$$p = \frac{s_{p1} * k_{p1}}{k_{p1} + [PR]} + \frac{s_{p2} * k_{p2}}{k_{p2} + [PD]}$$

$$\frac{d[OS]}{dt} = \mathrm{n} * \left(\frac{s_{1,2} * [OS]^2}{(k + [OS])^2 * dO * dS} + \frac{s_3 * [R]}{k + [R]} - dOS * [OS]\right) + \varphi(0, \sigma_{os}) * [OS]$$

$$\frac{d[N]}{dt} = \mathrm{n} * \left(\frac{s_4 * N^2}{k^2 + [N]^2 + p * [E]} + \frac{s_5 * [OS]}{k + [OS] + [Tcf3]} + \frac{s_6 * [R]}{k + [R]} - dN * [N]\right) + \varphi(0, \sigma_N) * [N]$$

$$\frac{d[M]}{dt} = \mathrm{n} * \left(\frac{s_7 * k}{k + [M]} + \frac{s_8 * k}{k + [N]} - dM * [M]\right) + \varphi(0, \sigma_M) * [M]$$

$$\frac{d[PR]}{dt} = \mathrm{n} * \left(\frac{s_{10} * k}{k + [OS]} + \frac{s_{11} * [N]}{k + [N]} + \frac{s_{12} * [M]}{k + [M]} - dPR * [PR]\right) + \varphi(0, \sigma_{PR}) * [PR]$$

$$\frac{d[R]}{dt} = \mathrm{n} * \left(\frac{s_{13} * [N]}{k + [N]} + \frac{s_{14} * k_r}{k_r + [B]} - dR * [R]\right) + \varphi(0, \sigma_R) * [R]$$

$$\frac{d[E]}{dt} = \mathrm{n} * \left(\frac{s_{15} * [OS]}{k + [OS]} - dE * [E]\right)$$

$$\frac{d[B]}{dt} = \mathrm{n} * \left(\alpha_1 - dB * \left(\frac{s_b * k_b}{k_b + [CH]}\right) * [B]\right) + \varphi(0, \sigma_B) * [B]$$

$$\frac{d[Tcf3]}{dt} = \mathrm{n} * \left(\alpha_2 - dTc * \left(1 + \frac{s_t * [B]}{k_t + [B]}\right) * [Tcf3]\right) + \varphi(0, \sigma_T) * [Tcf3]$$

N=Nanog; *OS*=Oct4-Sox2; *M*=Mycn; *PR*=Prdm14; *R*=Rest; *E*=Fgf4/Erk; *B*=β-catenin; *Tcf3*=Tcf3.

Parameter values (network in Figure S5a)

s_{p1}	30*	s_{14}	20*	**dM**	1#
s_{p2}	30*	s_{15}	2*	**dPR**	1#
k_{p1}	5	s_b	50*	**dR**	1#
k_{p2}	15	s_t	20*	**dE**	1#
$s_{1,2}$	75*	α_1	1*	**dB**	1#
s_3	1*	α_2	40*	**dTc**	1#
s_4	37*	**k**	10	σ_{OS}	0.02
s_5	2*			σ_N	0.05(0.04 for PD, Chiron=2)
s_6	0.01*	k_r	0.05	σ_M	0.02
s_7	10*	k_m	N/A	σ_{PR}	0.02
s_8	100*	k_t	10	σ_R	0.02
s_9	N/A	k_b	1	σ_B	0.02
s_{10}	15*	**dO**	1#	σ_T	0.02
s_{11}	30*	**dS**	1#	s_{nb}	10*
s_{12}	10*	**dOS**	1#	**n**	0.005
s_{13}	0.1*	**dN**	1#		

(* molecules/minute; # 1/minute)

Equations of the full system, with β-catenin activating Mycn (Network in Figure S6a)

$$p = \frac{s_{p1} * k_{p1}}{k_{p1} + [PR]} + \frac{s_{p2} * k_{p2}}{k_{p2} + [PD]}$$

$$\frac{d[OS]}{dt} = \mathrm{n} * \left(\frac{s_{1,2} * [OS]^2}{(k + [OS])^2 * dO * dS} + \frac{s_3 * [R]}{k + [R]} - dOS * [OS] \right) + \varphi(0, \sigma_{os}) * [OS]$$

$$\frac{d[N]}{dt} = \mathrm{n} * \left(\frac{s_4 * N^2}{k^2 + [N]^2 + p * [E]} + \frac{s_5 * [OS]}{k + [OS] + [Tcf3]} + \frac{s_6 * [R]}{k + [R]} - dN * [N] \right) + \varphi(0, \sigma_N) * [N]$$

$$\frac{d[M]}{dt} = \mathrm{n} * \left(\frac{s_7 * k}{k + [M]} + \frac{s_8 * k}{k + [N]} + \frac{s_9 * [B]}{k_m + [B]} - dM * [M] \right) + \varphi(0, \sigma_M) * [M]$$

$$\frac{d[PR]}{dt} = \mathrm{n} * \left(\frac{s_{10} * k}{k + [OS]} + \frac{s_{11} * [N]}{k + [N]} + \frac{s_{12} * [M]}{k + [M]} - dPR * [PR] \right) + \varphi(0, \sigma_{PR}) * [PR]$$

$$\frac{d[R]}{dt} = \mathrm{n} * \left(\frac{s_{13} * [N]}{k + [N]} + \frac{s_{14} * k_r}{k_r + [B]} - dR * [R] \right) + \varphi(0, \sigma_R) * [R]$$

$$\frac{d[E]}{dt} = \mathrm{n} * \left(\frac{s_{15} * [OS]}{k + [OS]} - dE * [E] \right)$$

$$\frac{d[B]}{dt} = \mathrm{n} * \left(\alpha_1 - dB * \left(\frac{s_b * k_b}{k_b + [CH]} \right) * [B] \right) + \varphi(0, \sigma_B) * [B]$$

$$\frac{d[Tcf3]}{dt} = \mathrm{n} * \left(\alpha_2 - dTc * \left(1 + \frac{s_t * [B]}{k_t + [B]} \right) * [Tcf3] \right) + \varphi(0, \sigma_T) * [Tcf3]$$

N=Nanog; *OS*=Oct4-Sox2; *M*=Mycn; *PR*=Prdm14; *R*=Rest; *E*=Fgf4/Erk; *B*=β-catenin; *Tcf3*=Tcf3.

Parameter values (network in Figure S6a)

s_{p1}	30*	s_{14}	20*	**dM**	1#
s_{p2}	30*	s_{15}	2*	**dPR**	1#
k_{p1}	5	s_b	50*	**dR**	1#
k_{p2}	15	s_t	20*	**dE**	1#
$s_{1,2}$	75*	α_1	1*	**dB**	1#
s_3	1*	α_2	40*	**dTc**	1#
s_4	37*	**k**	10	σ_{OS}	0.02
s_5	2*			σ_N	0.05(0.04 for PD, Chiron=2)
s_6	0.01*	k_r	0.05	σ_M	0.02
s_7	10*	k_m	0.05	σ_{PR}	0.02
s_8	100*	k_t	10	σ_R	0.02
s_9	20*	k_b	1	σ_B	0.02
s_{10}	15*	**dO**	1#	σ_T	0.02
s_{11}	30*	**dS**	1#	s_{nb}	10*
s_{12}	10*	**dOS**	1#	**n**	0.005
s_{13}	0.1*	**dN**	1#		

(* molecules/minute; # 1/minute)

Model simulations and bifurcation analysis

All SDE model simulations were run for 1000 cells, with parameters set at values reported above. Simulations in Figures 2a, 3a and Supplementary Figure S2a were performed 50 times; percentage of cells in the NH state, ± Standard Deviations, are provided in the main text. To compute numerical solutions of the SDEs, we used the Euler-Maruyama method. Stochastic simulations and density plots were implemented using the MATLAB platform (MathWorks). Steady state and bifurcation analysis (i.e. numerical continuation) was performed on the deterministic system (no noise), using the software xppaut (http://www.math.pitt.edu/$bard/xpp/xpp.html).

Agent-based simulations

The agent-based modelling involved creating a population of individual cells ('agents'), each of which has distinct characteristics including cell-cycle (lifetimes), cell fate (apoptosis/division), and cell states which depend on gene expression levels arising from the underlying GRN model. In our model, the state of each mESC is controlled by its intracellular expression of Nanog or Mycn.

Each cell's dynamics are governed by a set of SDEs that represent the GRN in Figure 1. The general setup of this model can be separated into three different compartments, each with their fixed models (Supplementary Figure S7). The SDE model of the GRN in each cell is solved throughout the simulation, which in turn influences the cell-cycle model. This cell-cycle model, governing when a cell will ultimately divide, feeds into a model that describes physical interactions between neighbouring cells, and also introduces new cells with associated GRN models into the simulation as division proceeds. The cell population is updated over discrete time steps, of duration Δt = 1 minute. At each time step the gene

expression levels are updated by integrating the SDE model. The current level of Nanog or Mycn in each cell defines the cell's 'state' and determines cell-cycle progression. In the Nanog-dependent cell-cycle model, cells can take Nanog High (NH) and Nanog Low (NL) states, as seen in the distributions in Figure 2a. In the Mycn-dependent cell-cycle model, the cells are either in a Mycn High (MH) or Mycn Low (ML) state. The distributions from which the cell-cycle durations are drawn are shown in Supplementary Figure S8, with the Nanog-dependent model allocated from Supplementary Figure S8a and the Mycn-dependent model allocated from Supplementary Figure S8b.

In our model, as in (1), cell-cycle duration is determined at the point of cell division, given the expression level of Nanog or Mycn. Each cell is also subject to a fixed probability of apoptosis, in order to match observed populations of 10% dead cells at any given point in the experiments (1), with dead cells remaining for 8 hours before being removed from the simulation, as fitted in (1).

The model was encoded in the Chaste modelling framework (2, 3), an extensive C++ library which allows for agent-based modelling of cell/tissue cultures. Within this model, we created an initial population of ~150 cells in a simulated culture that are initialised to their steady state values for each gene in serum/LIF or 2i/LIF (Figures 6a-d), or NH/NL states (Figure 6e). The mechanical model tracks the centre of each cell as a point, with cell neighbours defined using a Voronoi tessellation approach, and each pair of neighbouring cells connected by a linearly damped spring with a constant stiffness for each cell-cell connection (2). When a cell undergoes cell-division, the mechanical model introduced a new 'cell-centre' in a random direction from the centre of the mother cell. The Voronoi tessellation is then repeated to re-establish cell neighbours. The agent-based cell population is bounded by a layer of fixed non-proliferating cells to maintain a rigid square domain. The initial configuration of the cell-

population is a honeycomb mesh of equally spread cells, as shown in Supplementary Figure S9. Representative snapshots of agent-based model simulations are shown in Supplementary Figure S9, showing initial distribution of Nanog and Mycn levels within each cell (Supplementary Figures S9a, c) and mid-point examples during the simulation (Supplementary Figures S9b, d). Results in Figure 6a-d were run over 72 simulated hours, to compare with the experimental data-set.

Supplementary figure and table legends

Figure S1. Schematic of the process for identification of transcriptional interactions not present in the reference model (4)

Genes differentially expressed in serum/LIF vs 2i/LIF cultured mESCs (RNA-sequencing data from (5)) were analysed for Gene Ontology using the DAVID tool (6), and then filtered for specific Biological Processes (i.e. development and differentiation, proliferation, cell cycle, morphology and cell death). This is set '1' in the diagram. Genes reported in the mESC transcription factors compendium (CODEX (7)) being regulators of one of the core pluripotency network genes (Oct4, Sox2 or Nanog) were manually extracted, resulting in set '2' of the diagram. Genes in the shaded intersection of the two sets were considered for inclusion into the GRN. Interactions with β-catenin, identified from the data-set of genes differentially expressed between Chiron (indirect activator of β-catenin) and XAV (indirect inhibitor of β-catenin) (8) were included. Non-transcriptional interactions were retrieved from literature, and directions of interaction using NIA mouse ES cell bank (9). The number of genes identified or selected in each step is reported in the Figure. All data-sets are reported in Supplementary Table S1.

Figure S2. System dynamics and stability in 2i/LIF (PD=Chiron=20 AU, Nanog transcriptional noise not reduced)

(a) Steady-state distribution of Nanog (simulated in 1000 cells), showing that approximately 90% of the cells are in NH and 10% NL states. (b) Simulated time courses of Nanog and Oct4-Sox2 in 4 single cells over 15 hours. Oct4-Sox2 dimer concentration remains fairly constant compared to Nanog. The horizontal black dotted line represents the threshold between NH and NL. (c) Continuation of Nanog steady-state for the deterministic system (no

noise). Blue lines are stable steady-states, whilst the red line is unstable. Constant transcription rate, s_4 (dotted black line), intersects only the NH steady-state. Presence of NL cells in (a) is therefore only due to noise. (d) Sorting simulation; dynamics of 1000 NH or NL cells are simulated over 4 days, and the proportion of cells in the NH state is recorded every 12 hours (n=10, shaded areas indicate standard deviation). Cells can restore the steady-state distribution of approximately 90% NH and 10% NL.

Figure S3. System dynamics in serum/LIF and 2i/LIF upon Pdrm14 deletion

(a, b) Steady-state distribution of Nanog for the original GRN (Figure 1) and upon PRDM14 deletion in serum/LIF (a) and 2i/LIF (b, parameters set as in Figure 3).

Figure S4. System dynamics adding Nanog activation of β-catenin (PD=Chiron=0)

(a-d) Steady-state distributions in serum/LIF of Nanog (a, c) and β-catenin (b, d) without (a, b) and with (c, d) Nanog activation of β-catenin. (e-h) Steady-state distributions in 2i/LIF (parameters set as in Figure 3) of Nanog (e, g) and β-catenin (f, h) without (e, f) and with (g, h) Nanog activation of β-catenin.

Figure S5. System dynamics removing β-catenin regulation of Mycn

(a) Network diagram, with no inhibition of Mycn by β-catenin. (b) Continuation of Nanog steady-state for the deterministic system with no interaction between β-catenin and Mycn. Serum/LIF parameters used (PD=0, Chiron=0). Blue lines are stable steady-states, whilst the red line is unstable. Constant transcription rate, s_4 (dotted black line), intersects both stable steady-states. (c) Steady-state distribution of Nanog for serum/LIF parameters, showing approximately 50% of cells in NH and NL states. (d) Continuation of Nanog steady-state for 2i/LIF parameters (PD=2, Chiron=2). Blue lines are stable steady-states, whilst the red line is unstable. Constant transcription rate, s_4 (dotted black line), is very close to the saddle-node

bifurcation point. (e) Steady-state distribution of Nanog for 2i/LIF parameters, showing approximately 90% of cells in NH and 10% in NL states. (f) Two-parameter continuation (PD and Chiron). The area above the curve denotes the parameter region in which the deterministic system presents monostability (NH only).

Figure S6. System dynamics with β-catenin activating Mycn rather than inhibiting it

(a) Network diagram, with activation of Mycn by β-catenin. (b) Continuation of Nanog steady-state for deterministic system with β-catenin activating Mycn. Serum/LIF parameters used (PD=0, Chiron=0). Blue lines are stable steady-states, whilst the red line is unstable. Constant transcription rate, s_4 (dotted black line), intersects both stable steady-states. (c) Steady-state distribution of Nanog for serum/LIF parameters, showing approximately 50% of cells in NH and NL states. (d) Continuation of Nanog steady-state for 2i/LIF parameters (PD=2, Chiron=2). Blue lines are stable steady-states, whilst the red line is unstable. Constant transcription rate, s_4 (dotted black line), is very close to the saddle-node bifurcation point. (e) Steady-state distribution of Nanog for 2i/LIF parameters, showing approximately 90% of cells in NH and 10% in NL states. (f) Two-parameter continuation (PD and Chiron). The area above the curve denotes the parameter region in which the deterministic system presents monostability (NH only).

Figure S7. Modular setup of the agent-based model and Nanog distributions in agent-based simulations

(a) The agent-based model can be thought of as being made up of three separate interconnected modules. The first (1) is the gene-regulatory network, described by SDEs. The role of the agent-based model is to add the effects that this module has on the cell-cycle and mechanics of cells in a population. The state of genes within each cell affects the lengths of

the cell-cycles, and thus at what point a cell divides, which is then managed by the mechanical model which introduces new cells into the simulation which requires the creation of new gene networks for each cell. (b) Nanog distribution in the agent-based cell population at the end of day 4 of simulation in Serum/LIF when using a Nanog-based cell-cycle model, showing a ~50/50 split of NH and NL as expected from the non-agent based simulation. (c) Nanog distribution in the agent-based cell population at the end of day 4 of simulation in 2i/LIF when using a Nanog-based cell-cycle model, showing the higher proportion of NH than in the Serum/LIF medium. (d) Nanog distribution in the agent-based cell population at the end of day 4 of simulation in Serum/LIF when using a Mycn-based cell-cycle model, showing a ~50/50 split of NH and NL. (e) Nanog distribution in the agent-based cell population at the end of day 4 of simulation in 2i/LIF medium when using a Mycn-based cell-cycle model, showing the increase in proportion of NH cells, with a residual population of NL cells remaining.

Figure S8. Cell-cycle model distributions for each state of gene concentration.

(a, b) We allocate cell-cycle lengths based on whether a cell is in the NH or NL state in the Nanog-dependent cell-cycle model (a), or in MH or ML state in the Mycn-dependent cell-cycle model (b). In the main text, fitted values are reported for the mean of cell-cycle length (normal distribution, standard deviation of 1 hour).

Figure S9. Snapshots of agent-based simulations.

(a, c) Initial set-up of agent-based simulations in serum/LIF; Nanog (a) and Mycn (c) expression is indicated; the distribution is ~50/50 NH and NL. (b, d) A 'mid' simulation snapshot, showing the growing number of cells within the population, and levels of Nanog (b) and Mycn (d).

Supplementary Table S1

Genes differentially expressed in 2i/LIF vs serum/LIF from (5) (as in the paper, p value < 0.2, fold change> 2). Gene Ontology analysis (biological processes reported, analysed with DAVID (6). CODEX TFs (7), with existence of interaction shown with a tick. Gene Ontology analysis including intersection with CODEX and Biological Processes categorized. List of all NIA bank genes (9). Interactions of CODEX and NIA bank (p-value<0.05) considered for final network. List of genes differentially expressed between Chiron (Wnt pathway activator through inhibition of Gsk3) and XAV (tankyrase inhibitor antagonizing Wnt signaling through stabilisation of the Axin2 inhibitor) treated mESCs, from (8).

References

1. Herberg M, Glauche I, Zerjatke T, Winzi M, Buchholz F, Roeder I. Dissecting mechanisms of mouse embryonic stem cells heterogeneity through a model-based analysis of transcription factor dynamics. Journal of the Royal Society Interface. 2016;13(117).

2. Mirams GR, Arthurs CJ, Bernabeu MO, Bordas R, Cooper J, Corrias A, et al. Chaste: an open source C++ library for computational physiology and biology. PLoS Comput Biol. 2013;9(3):e1002970.

3. Osborne JM, Fletcher AG, Pitt-Francis JM, Maini PK, Gavaghan DJ (2017) Comparing individual-based approaches to modelling the self-organization of multicellular tissues. PLoS Comput Biol 13(2): e1005387. doi:10.1371/journal.pcbi.1005387

4. Herberg M, Kalkan T, Glauche I, Smith A, Roeder I. A model-based analysis of culture-dependent phenotypes of mESCs. PLoS One. 2014;9(3):e92496.

5. Marks H, Kalkan T, Menafra R, Denissov S, Jones K, Hofemeister H, et al. The transcriptional and epigenomic foundations of ground state pluripotency. Cell. 2012;149(3):590-604.

6. Huang DW, Sherman BT, Tan Q, Kir J, Liu D, Bryant D, et al. DAVID Bioinformatics

Resources: expanded annotation database and novel algorithms to better extract biology from large gene lists. Nucleic Acids Research. 2007;35(Web Server issue):W169-75.

7. Sanchez-Castillo M, Ruau D, Wilkinson AC, Ng FS, Hannah R, Diamanti E, et al. CODEX: a next-generation sequencing experiment database for the haematopoietic and embryonic stem cell communities. Nucleic Acids Research. 2015;43(Database issue):D1117-23.

8. Zhang X, Peterson KA, Liu XS, McMahon AP, Ohba S. Gene regulatory networks mediating canonical Wnt signal-directed control of pluripotency and differentiation in embryo stem cells. Stem Cells. 2013;31(12):2667-79.

9. Nishiyama A, Xin L, Sharov AA, Thomas M, Mowrer G, Meyers E, et al. Uncovering early response of gene regulatory networks in ESCs by systematic induction of transcription factors. Cell Stem Cell. 2009;5(4):420-33.

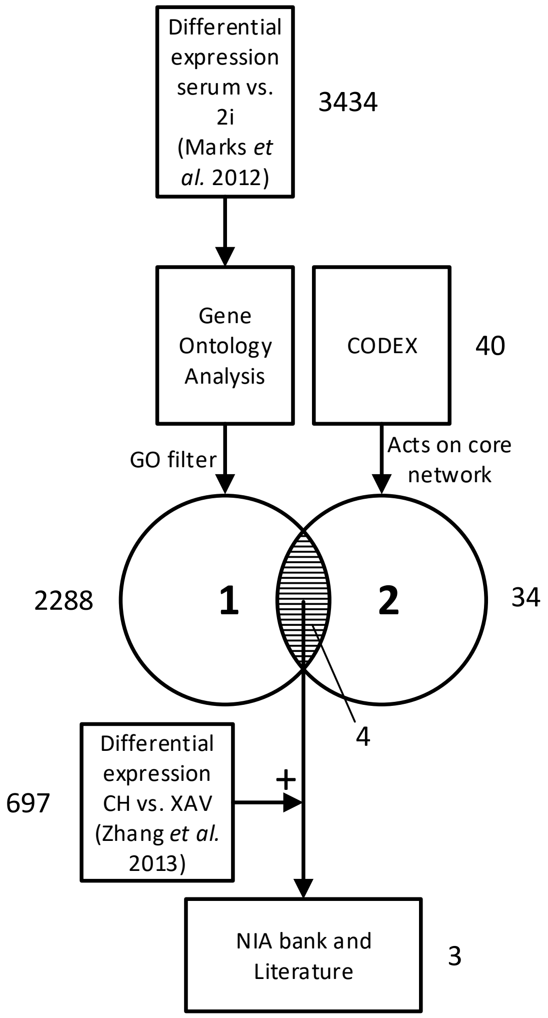
Differential expression serum vs. 2i (Marks et al. 2012)
3434
Gene Ontology Analysis
CODEX
40
GO filter
Acts on core network
2288
1
2
34
4
Differential expression CH vs. XAV (Zhang et al. 2013)
697
+
NIA bank and Literature
3

Figure S1

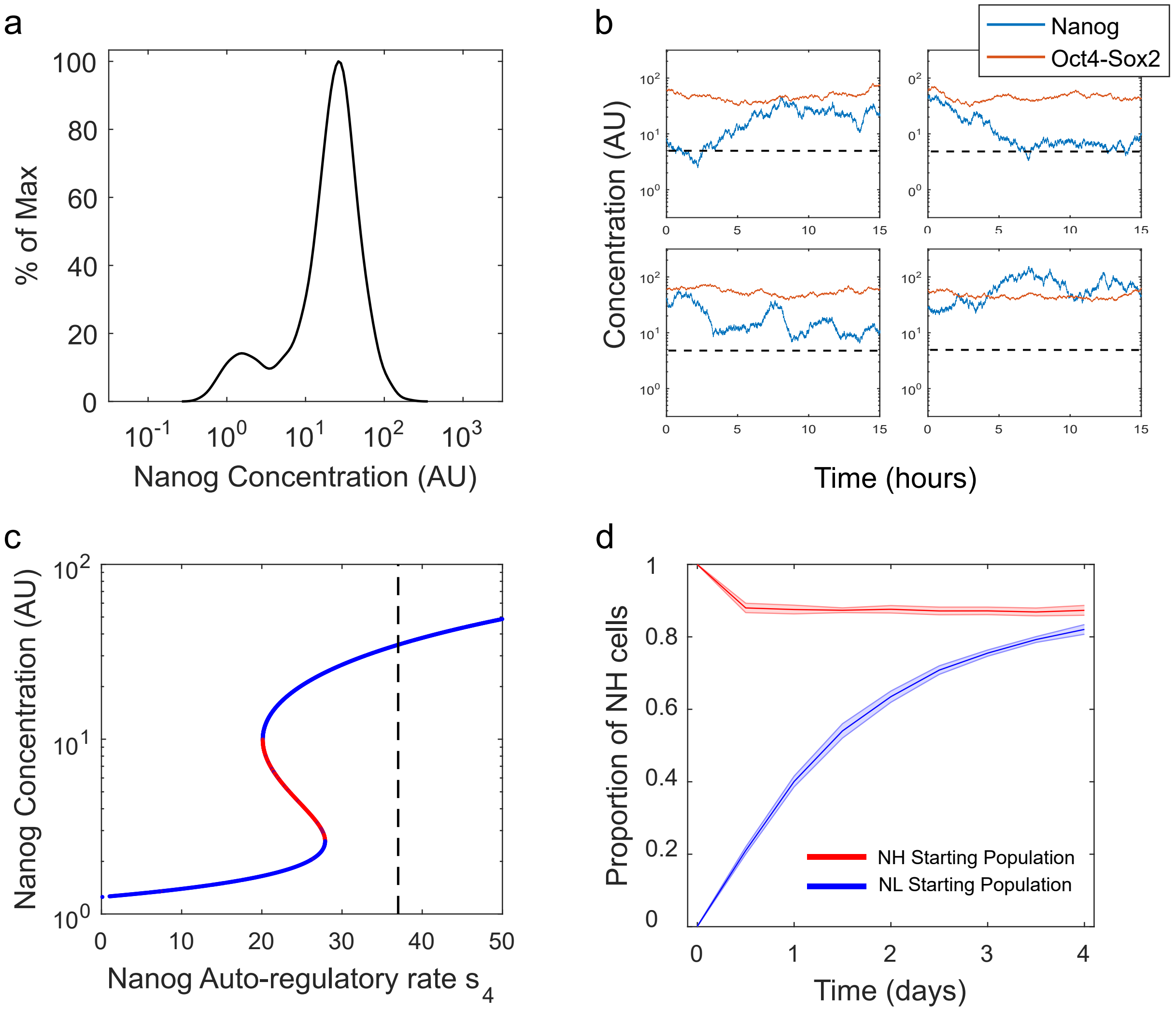

a
% of Max
Nanog Concentration (AU)
b
Nanog
Oct4-Sox2
Concentration (AU)
Time (hours)
c
Nanog Concentration (AU)
Nanog Auto-regulatory rate s_4
d
Proportion of NH cells
NH Starting Population
NL Starting Population
Time (days)

Figure S2

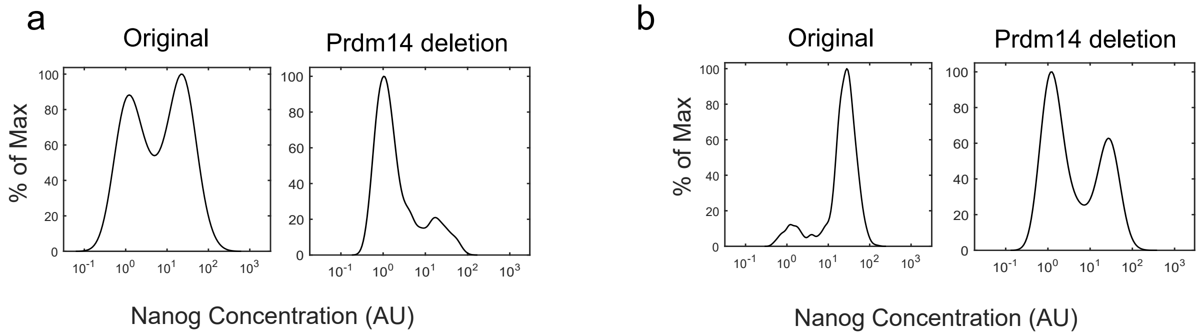
a
Original
Prdm14 deletion
% of Max
Nanog Concentration (AU)
b
Original
Prdm14 deletion
% of Max
Nanog Concentration (AU)

Figure S3

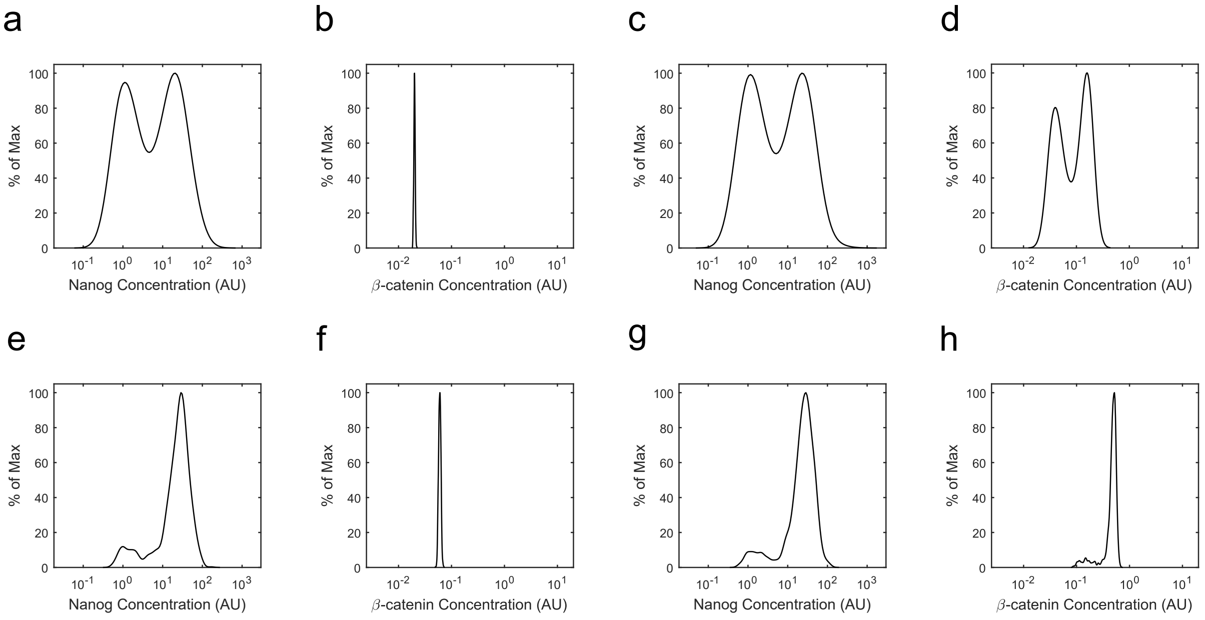

a
b
c
d
e
f
g
h
% of Max
Nanog Concentration (AU)
β-catenin Concentration (AU)

Figure S4

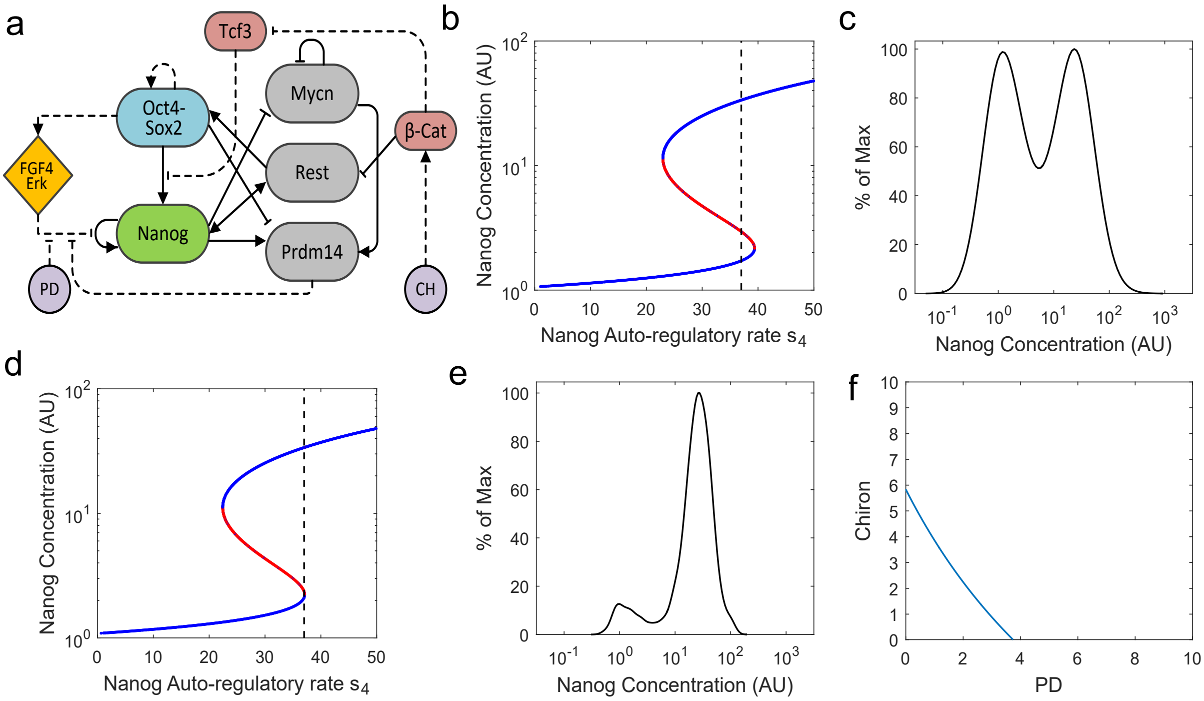

a
Tcf3
Mycn
Oct4-Sox2
FGF4 Erk
Rest
β-Cat
Nanog
Prdm14
PD
CH
b
Nanog Concentration (AU)
Nanog Auto-regulatory rate s_4
c
% of Max
Nanog Concentration (AU)
d
Nanog Concentration (AU)
Nanog Auto-regulatory rate s_4
e
% of Max
Nanog Concentration (AU)
f
Chiron
PD

Figure S5

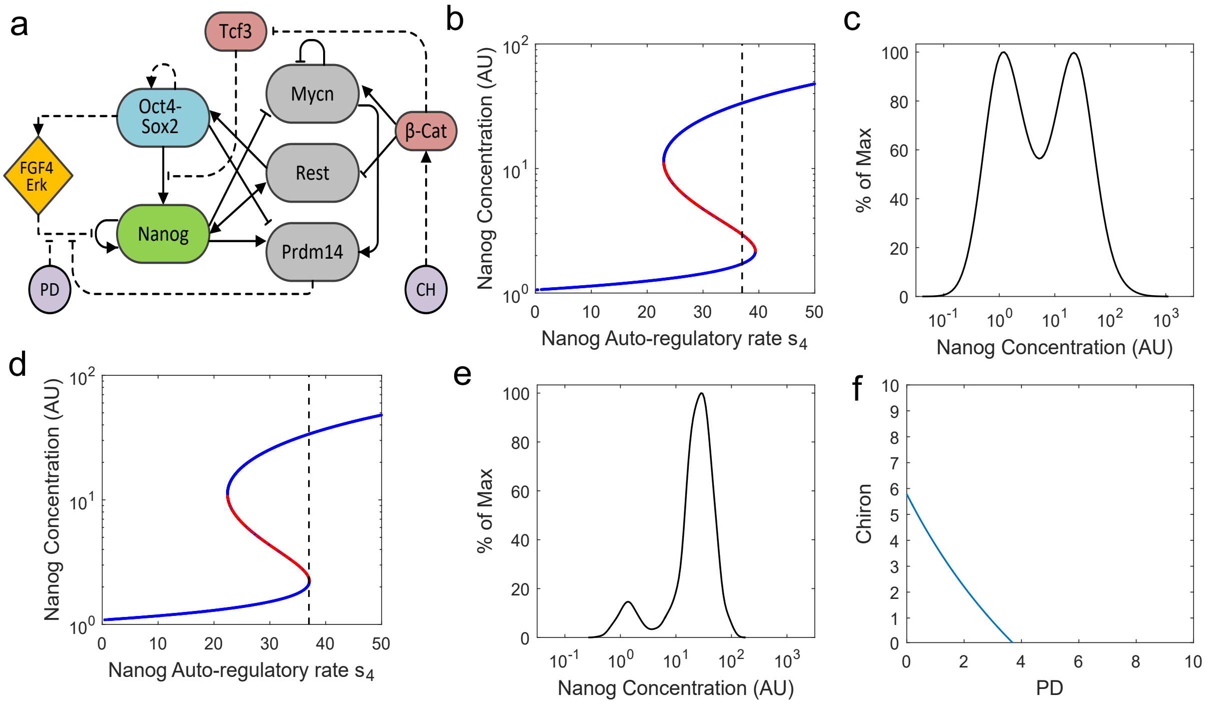
a
Tcf3
Mycn
Oct4-
Sox2
FGF4
Erk
Rest
β-Cat
Nanog
Prdm14
PD
CH
b
Nanog Concentration (AU)
Nanog Auto-regulatory rate s4
c
% of Max
Nanog Concentration (AU)
d
Nanog Concentration (AU)
Nanog Auto-regulatory rate s4
e
% of Max
Nanog Concentration (AU)
f
Chiron
PD

Figure S6

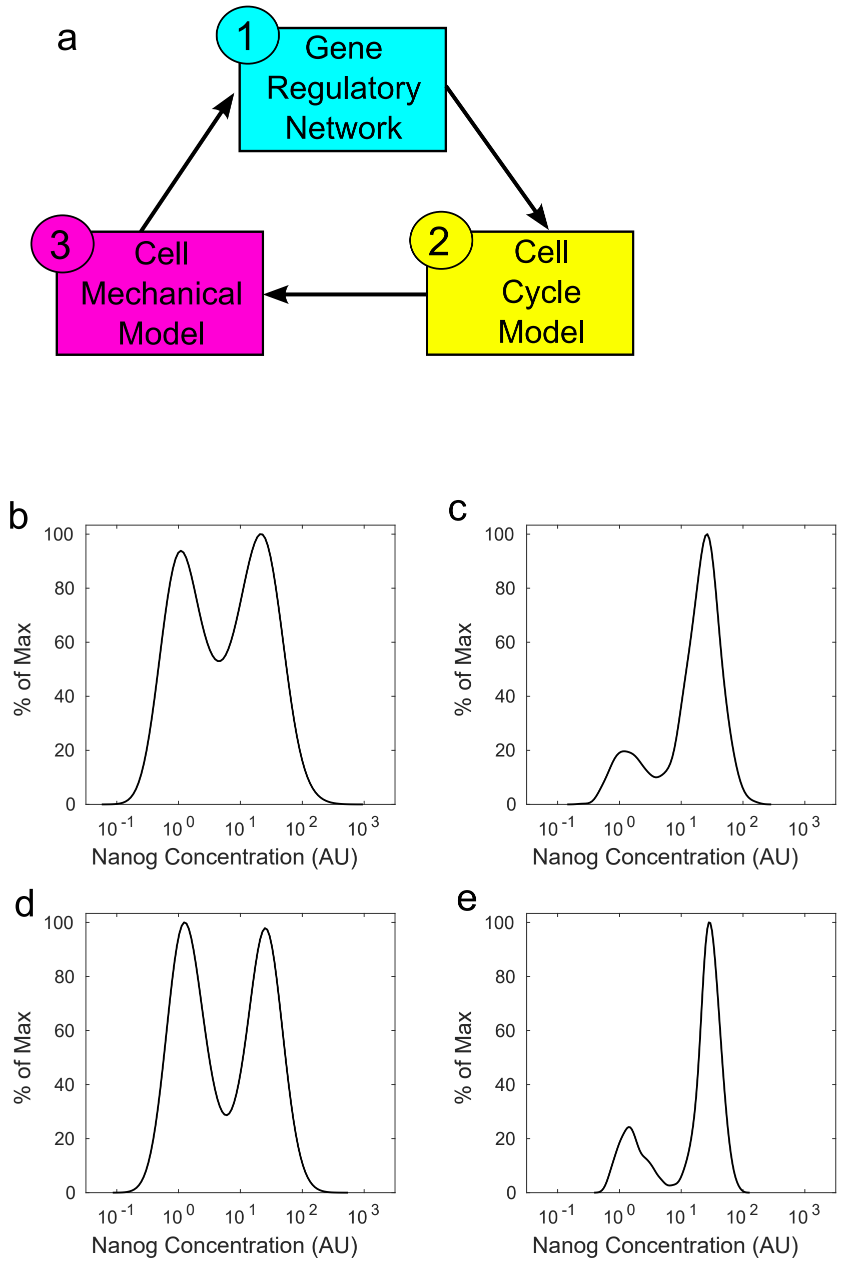
a
1
Gene Regulatory Network
2
Cell Cycle Model
3
Cell Mechanical Model
b
c
d
e
100
80
60
40
20
0
% of Max
10^{-1}
10^{0}
10^{1}
10^{2}
10^{3}
Nanog Concentration (AU)

Figure S7

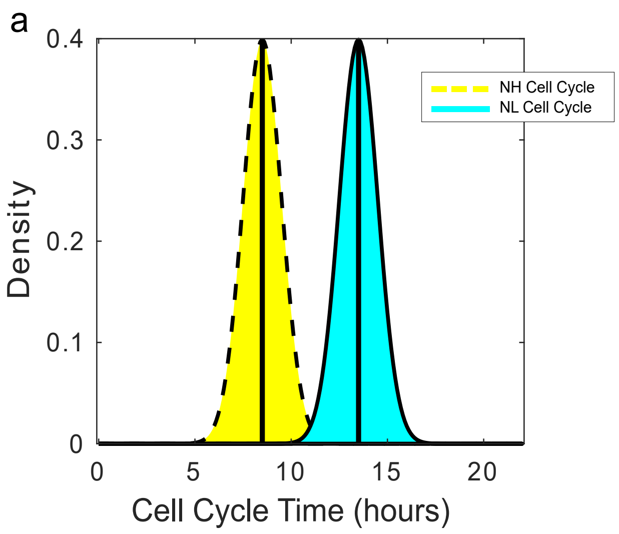
a
b
0.4
0.3
0.2
0.1
0
Density
0
5
10
15
20
Cell Cycle Time (hours)
NH Cell Cycle
NL Cell Cycle
MH Cell Cycle
ML Cell Cycle

Figure S8

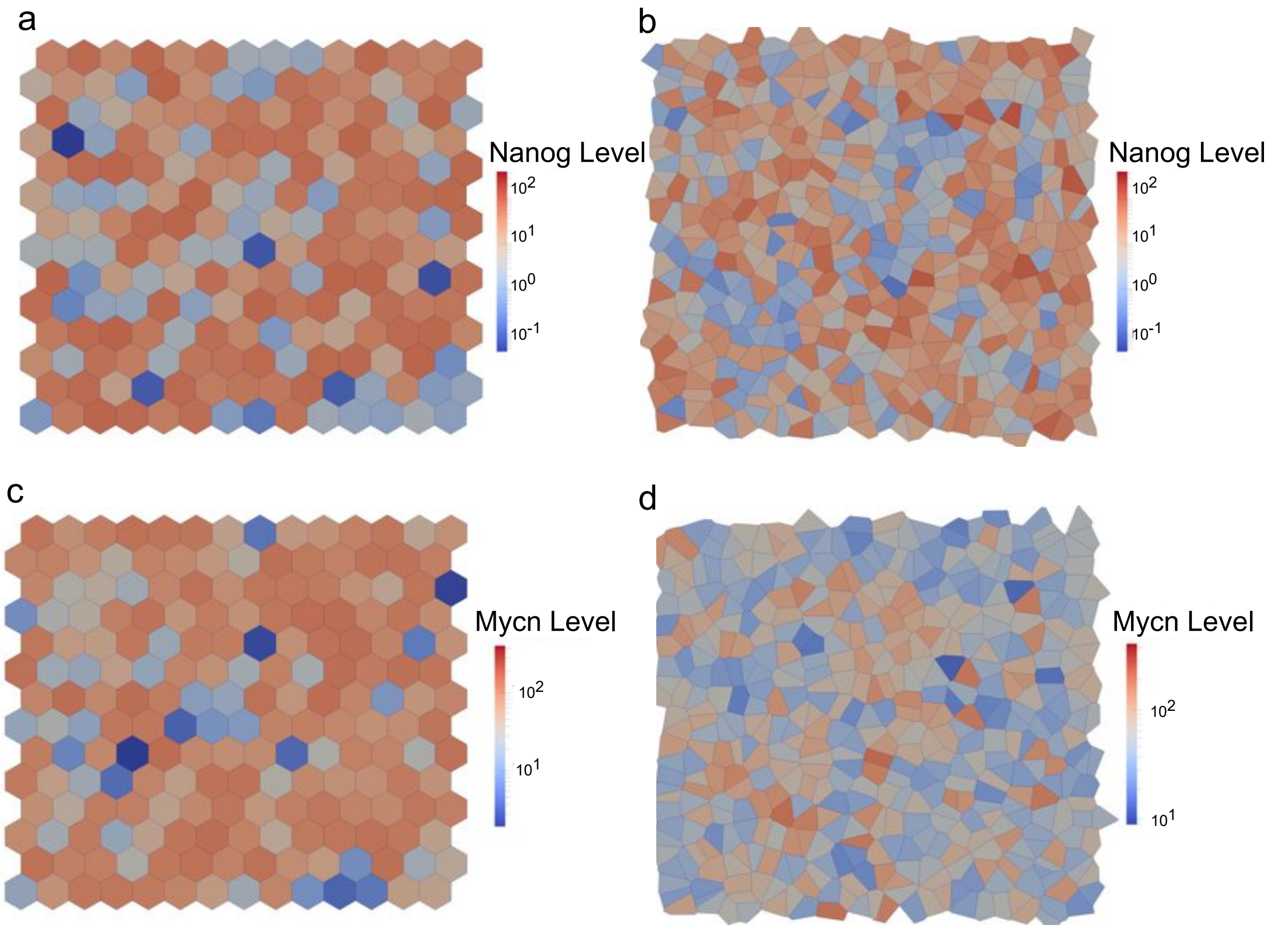
a
b
c
d
Nanog Level
10^2
10^1
10^0
10^{-1}
Nanog Level
10^2
10^1
10^0
10^{-1}
Mycn Level
10^2
10^1
Mycn Level
10^2
10^1

Figure S9
